# Can Sirtuin 1 Serve as a Therapeutic Target in Pulmonary Arterial Hypertension? A Comprehensive Review

**DOI:** 10.3390/molecules30183740

**Published:** 2025-09-15

**Authors:** Sandra Budziak, Monika Kloza, Anna Krzyżewska, Marta Baranowska-Kuczko

**Affiliations:** 1 Department of Experimental Physiology and Pathophysiology, Medical University of Białystok, 15-222 Białystok, Poland; 41638@student.umb.edu.pl (S.B.); anna.krzyzewska@umb.edu.pl (A.K.); 2 Department of Clinical Pharmacy, Medical University of Białystok, 15-222 Białystok, Poland

**Keywords:** pulmonary hypertension, pulmonary arterial hypertension, sirtuin 1, pulmonary artery vascular smooth muscle cells, pulmonary artery endothelial cells, vascular remodeling, proliferation, inflammation, oxidative stress, mitochondrial biogenesis

## Abstract

Pulmonary arterial hypertension (PAH) is a progressive, currently incurable disease characterized by elevated pulmonary arterial pressure, vascular remodeling, and right ventricular hypertrophy, eventually leading to heart failure and death. Sirtuin 1 (SIRT1), a NAD^+^-dependent deacetylase, regulates endothelial and vascular smooth muscle function, and its activation by compounds such as resveratrol or SRT1720 shows therapeutic potential by reducing pulmonary and right ventricular pressures and limiting vascular remodeling in both preventive and therapeutic experimental models, highlighting their potential translational relevance. To date, no comprehensive review has focused on the role of SIRT1 in PAH. This review summarizes the molecular mechanisms of SIRT1 action in the cardiopulmonary system and discusses its therapeutic potential in PAH treatment.

## 1. Introduction

Pulmonary hypertension (PH) is defined by mean pulmonary arterial pressure (mPAP) over 20 mmHg, confirmed by right-sided heart catheterization. The updated guidelines classify PH into five groups: pulmonary arterial hypertension (PAH), PH associated with left heart disease, PH associated with lung diseases and/or hypoxia, PH associated with pulmonary artery (PA) obstructions, and PH with unclear and/or multifactorial mechanisms [1]. Detailed hemodynamic categorization into pre-capillary PH, isolated post-capillary PH, or combined pre- and post-capillary PH is determined based on pulmonary vascular resistance and pulmonary arterial wedge pressure values [1]. Despite progress in the therapeutic strategies, the prognosis for PH remains poor, highlighting the importance of early detection and a deeper understanding of the molecular mechanisms underlying the disease to enable the development of more effective, targeted therapies and treatment approaches [1,2]. Group I (PAH) currently represents one of the greatest clinical challenges as it is the rarest type in which profound changes in the PA structure and function are the key problem and main therapeutic target [1]. The lesions manifest as excessive vasocontraction and remodeling of the distal pulmonary arterioles with the formation of characteristic plexiform lesions, resulting in increased vascular resistance in the pulmonary circulation and consequently leading to excessive right ventricular (RV) afterload. A number of global registers have shown that women develop PAH approximately 2.3 times more often than men. Paradoxically, however, despite the higher incidence, the clinical course of PAH in women is characterized by a milder profile, which is probably related to the protective effect of estrogens on the right ventricle [2]. Available therapies primarily focus on promoting pulmonary vasorelaxation and modulating the nitric oxide (NO), endothelin-1 (ET-1), and prostacyclin (PGI2) pathways, while other molecular targets of the disease remain beyond the reach of therapeutic intervention. Imbalanced signaling by the transforming growth factor-beta (TGF-β) superfamily significantly contributes to dysregulated vascular cell proliferation in PAH, characterized by overactive pro-proliferative mothers against decapentaplegic homolog 2/3 (Smad2/3) signaling occurring concurrently with deficient antiproliferative mothers against decapentaplegic homolog 1/5/8 (Smad1/5/8) signaling. This dysregulation, along with the interplay of other pathways, such as bone morphogenetic protein (BMP) signaling and the phosphoinositide 3-kinase/protein kinase B (PI3K/Akt) pathway, plays a pivotal role in the pathological remodeling of the pulmonary vasculature and the progression of PAH. The most recent breakthrough in PAH therapy is sotatercept, an activin receptor type IIA fusion protein (IIA-Fc) that restores the imbalance between overpromoted Smad2/3 pro-proliferative signaling and Smad1/5/8 antiproliferative signaling in PAH patients [3]. Following promising clinical trial results, sotatercept was approved in 2024 by the U.S. Food and Drug Administration (FDA) and the European Medicines Agency (EMA) as a supplemental agent to conventional PAH treatment. These findings support the ongoing search for treatments targeting alternative disease mechanisms—such as vascular remodeling, oxidative stress, and inflammation—which will be discussed later in this review [4].

Among emerging regulators of vascular homeostasis, sirtuin 1 (SIRT1) has garnered significant attention. In the cardiovascular system and beyond, SIRT1—a nicotinamide adenine dinucleotide (NAD^+^)-dependent deacetylase—plays a pivotal role in regulating key cellular processes such as inflammation, oxidative stress, metabolism, and apoptosis [5,6]. Notably, dysregulation of these processes is central to the pathogenesis of PAH, particularly in the context of pulmonary vascular remodeling, endothelial dysfunction, and excessive proliferation of pulmonary artery smooth muscle cells (PASMCs). Recent evidence suggests that SIRT1 activation exerts protective effects in experimental models of PH by mitigating oxidative injury, restoring endothelial integrity, and inhibiting maladaptive vascular remodeling [7,8]. These findings highlight a compelling link between SIRT1 activity and the modulation of key pathophysiological pathways in PH.

To date, no comprehensive review has specifically addressed the role of SIRT1 in PH/PAH. This review aims to provide a comprehensive overview of current knowledge regarding the role of SIRT1 in the pathophysiology of PH, summarizing its molecular mechanisms of action and evaluating its potential as a therapeutic intervention in this challenging clinical condition. By integrating findings from preclinical studies with a particular emphasis on group 1 PH, we aim to shed light on the promise of SIRT1 as a novel target in the treatment of PAH.

In this paper, although most experimental models are intended to mimic PAH, the term “PAH” refers specifically to the human condition, and “PH” is used to refer to the experimental models. This is dictated by the slight differences between experimental PH and the clinical presentation of this disease entity. For clarity and summarization, we will use “pulmonary (arterial) hypertension” to represent findings from both human studies and experimental models.

## 2. Molecular and Cellular Pathophysiology of Pulmonary (Arterial) Hypertension

PAH is a rare and incurable pulmonary vascular disease with a complex and not yet fully understood etiopathogenesis. It is presumed that pulmonary vascular endothelial dysfunction and changes in the microenvironment may be among the key steps that initiate the disease process [9,10]. Genetic factors, hypoxia, drugs and toxins, and shear stress, as well as inflammation and oxidative stress, can trigger endothelial cell dysfunction and change its phenotype to pro-proliferative, pro-inflammatory and prothrombotic [9,11]. Although PAH is divided into several subtypes due to its etiology (e.g., idiopathic pulmonary arterial hypertension (iPAH), PAH associated with connective tissue disease (PAH-CTD), or hereditary PAH) [1], most of these subtypes involve dysregulation of similar signaling pathways in PA cells, resulting in their excessive proliferation and remodeling of distal pulmonary arterioles [12]. Obstruction of the pulmonary arterioles and the associated increase in resistance in the pulmonary circulation can transmit to the RV, resulting in heart failure and premature death of patients [13]. Beyond structural changes in the pulmonary vasculature, disease progression is driven by a network of proliferative, inflammatory, oxidative, metabolic, and thrombotic mechanisms [2,14,15].

In PAH, excessive proliferation and apoptosis resistance of PASMCs are driven by overproduction of growth factors such as platelet-derived growth factor (PDGF) and TGF-β, which activate pro-survival and pro-proliferative signaling pathways, including mechanistic target of rapamycin (mTOR) and Akt [2,7,9,11,13,14,15,16]. As the disease progresses, PASMCs adopt a secretory, hyperproliferative, and apoptosis-resistant phenotype. These coactivators promote cell proliferation by influencing gene expression related to cell cycle progression and survival. Additionally, the mTOR complex 1 (mTORC1) and complex 2 (mTORC2) play pivotal roles in mediating growth-promoting signals. The activation of the mTOR pathway, along with Akt signaling, enhances protein synthesis and cell growth, further supporting the proliferative phenotype of PASMCs. Transcription factors like hypoxia-inducible factors (HIFs) and nuclear factor erythroid 2-related factor 2 (Nrf2) further support this state by enhancing cellular responses to hypoxia and oxidative stress, contributing to vascular remodeling and disease progression [7,9,10,15,16]. Nrf2 enhances PASMC survival and proliferation by upregulating antioxidant gene expression, reducing oxidative stress and inflammation. HIFs drive PASMC proliferation under low-oxygen conditions by regulating genes involved in angiogenesis, metabolism, and cell survival. In PAH, both Nrf2 and HIF pathways are upregulated, reinforcing the vascular remodeling that characterizes disease progression.

Inflammation has been shown to promote vascular smooth muscle cell (VSMC) proliferation and extracellular matrix deposition, resulting in thickening, decreased compliance, and remodeling of blood vessel walls in PAH [17]. In animal models [18], as well as in lung biopsy specimens from patients with PAH [19,20,21], inflammatory cells, including T and B lymphocytes, macrophages, dendritic cells, mast cells, or neutrophils, have been shown to form perivascular inflammatory infiltrates in fragments involving remodeled vessels. In addition, it has been shown that levels of certain inflammatory cytokines and chemokines, including tumor necrosis factor-alpha (TNF-α), interferon-gamma (IFN-γ), interleukin-1beta (IL-1β), interleukin-2 (IL-2), interleukin-4 (IL-4), interleukin-6 (IL-6), interleukin-18 (IL-18), interleukin-10 (IL-10), matrix metalloproteinase 9 (MMP9), CC Motif Chemokine Ligand 5 (CCL5), or monocyte chemotactic protein 1 (MCP-1), are elevated in patients with PAH and may correlate with disease severity, and thus monitoring their changes may be diagnostically useful in patients with PAH [18,22]. It is presumed that therapies targeting inflammatory pathways in PAH may have positive results and reduce disease progression.

In addition to inflammation, oxidative stress is closely associated with endothelial dysfunction and contributes to reduced NO bioavailability and increased synthesis of endothelium-derived vasoconstrictive factors, i.e., ET-1 [15]. In addition, nicotinamide adenine dinucleotide phosphate (NADPH) oxidase 4 (NOX4)-derived reactive oxygen species (ROS) have been shown to promote the activation of a number of growth factors and promote a change in endothelial cell phenotype to mesenchymal via the TGF-β pathway and have also been implicated in the proliferation of PAH PASMCs [23], which may have been associated with dysregulation of the Kelch-like ECH-associated protein 1 (Keap-1)/Nrf2 pathway [15].

Mitochondrial dysfunction is one of the key contributors to the PAH development and not only affects energy metabolism but also triggers a cascade of events that lead to inflammatory responses, vascular remodeling, and altered hypoxic responses, thereby representing a central mechanism in the progression of the disease. In PAH, metabolic pathways are significantly disrupted, as exemplified by the Warburg effect. This phenomenon, initially identified in cancer cells, has also been observed in pulmonary artery endothelial cells (PAECs) and PASMCs of patients with iPAH [24]. Mitochondria in pulmonary vascular cells and the RV exhibit decreased oxidative phosphorylation alongside increased aerobic glycolysis. As respiratory activity diminishes, hypoxia-inducible factors alpha (HIF-α) become transcriptionally upregulated and stabilized, promoting a shift towards glycolysis. This shift leads to the production of NADPH, which serves as a cofactor for NOX enzymes that generate ROS. The oxidative stress mediated by NOX can worsen endothelial dysfunction, leading to a shift in the endothelial phenotype towards a pro-constrictive state. In PAH, endothelial cell mitochondria display a fission phenotype, resulting in fragmentation. HIF-1α regulates dynamin-related protein 1 (Drp1), contributing to excessive proliferation of PASMCs [25]. Upon mitochondrial damage, phosphatase and tensin homologue deleted on chromosome 10 (PTEN)-induced putative kinase 1 (PINK1) activates E3 ubiquitin–protein ligase Parkin, facilitating mitophagy. FUNDC1, a receptor involved in this process, promotes PASMC proliferation and pulmonary vascular remodeling by upregulating HIF-1α [26].

This metabolic alteration in pulmonary vascular cells drives extensive vascular remodeling, occluding the small PA, ultimately resulting in increased pulmonary vascular resistance and elevated PAP. Collectively, these changes highlight the critical role of metabolic reprogramming and oxidative stress in the progression of PAH in humans and in experimental models [27,28,29]. This metabolic shift is also maintained through abnormal activation of transcription factors like cellular myelocytomatosis oncogene (c-Myc), along with suppression of the Forkhead box protein O1 (FOXO1) [7].

## 3. Sirtuin 1

Sirtuin 1, the homolog 1 of the yeast silent information regulator (Sir2), stands out by garnering extensive research attention as one of the seven subtypes of the sirtuin family. It is a NAD^+^-dependent histone deacetylase, helping cells with growth and division [30]. The primary function of SIRT1 involves the removal of acetyl groups from lysine side chains [31], which contributes to controlling gene transcription and therefore regulating numerous physiological functions, from metabolism [32,33] to stress reactions [34].

**Figure 1:**
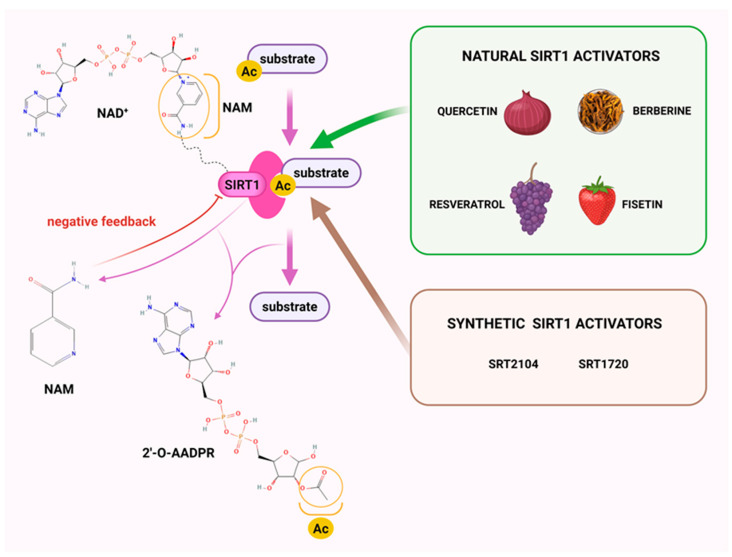
Deacetylation mechanism by sirtuin 1 (SIRT1) and its activators. 2′-*O*-AADPR–2′-*O*-acetyl-ADP-ribose, Ac—acetyl group, NAD^+^—nicotinamide adenine nucleotide, NAM—nicotinamide. Created in BioRender. Krzyżewska, A. (2025) https://BioRender.com/hih4gjx (accessed on 21 August 2025).

SIRT1 activity is regulated by the NAD^+^/NADH ratio [35,36], which can be increased directly by adenosine monophosphate-activated kinase (AMPK) and indirectly via AMPK activators. Calorie restriction (CR), fasting, and exercise, which limit energy and reduce adenosine triphosphate (ATP) levels, also elevate NAD^+^ levels through AMPK activation [37]. SIRT1 recognizes NAD^+^ at active sites on the Rossmann fold and deacetylates substrates by transferring the acetyl group to the adenosine diphosphate (ADP)-ribosyl part of NAD^+^, yielding 2′-*O*-acetyl-ADP-ribose and nicotinamide (NAM). The activity of SIRT1 is negatively regulated by NAM through feedback inhibition [38] (Figure 1).

SIRT1 is expressed throughout the body, including vasculature, where it is present in endothelial cells (ECs) [39,40,41,42,43], VSMCs of human subcutaneous microvasculature [43,44], popliteal arteries [45], internal mammary artery, aorta, and saphenous vein [46], mice plaques [46], aortas [47,48,49], small mesenteric arteries [49] and perivascular adipose tissues [41], adventitia [43], and cardiomyocytes [50,51,52,53,54,55]. Predominantly located in the nucleus, SIRT1 deacetylates histones and transcription factors, influencing glucose and lipid metabolism, cellular aging, endothelial and smooth muscle cell function, inflammation, oxidative stress, and extracellular matrix degradation [33,42,56,57,58]. It provides protective effects on the cardiovascular system by boosting antioxidant and anti-inflammatory defenses through the upregulation of antioxidant genes, such as Forkhead box protein O3a (FOXO3a) and superoxide dismutase 2 (SOD2) [59], and suppression of pro-inflammatory gene transcription, including nuclear factor-kappa B (NF-κB) [56].

Extensive research underscores the pivotal role of SIRT1 in the aging process, which is marked by progressive structural and functional decline of tissues and the emergence of age-associated phenotypic alterations. SIRT1 expression is notably reduced in older individuals, whereas its overexpression has been shown to confer protective effects against age-related diseases [60]. SIRT1 activates AMPK, leading to the inhibition of the mTOR signaling pathway and induction of autophagy, a mechanism recognized for its role in delaying aging. Furthermore, SIRT1-mediated deacetylation of FOXO1 is reported to significantly contribute to the regulation of autophagic processes [61].

Beyond senescence, SIRT1 modulates key cellular processes, including proliferation, differentiation, apoptosis, and fibrosis, partly via regulation of TGF-β1, a critical cytokine secreted by ECs and PASMCs, through the TGF-β1/Smad3 signaling axis [62,63]. In addition, SIRT1 deacetylates a tumor suppressor protein (p53), which results in the inhibition of its pro-apoptotic function [61,64].

SIRT1 also exerts anti-inflammatory and vasoprotective effects by suppressing NF-κB signaling, thereby reducing the levels of inflammatory cytokines (such as TNF-α and IL-6) released from VSMCs, which limits ET-1-mediated vasoconstriction [65], and decreasing the activation of phosphorylated Akt (p-Akt)—a component of the Akt/Nrf2/NF-κB signaling cascade [66]. Furthermore, SIRT1 inhibits NF-κB-driven tissue factor activation, contributing to protection against arterial thrombosis [67]. In addition, SIRT1 enhances the synthesis and bioavailability of NO, the principal endothelium-derived vasodilator, via deacetylation and activation of endothelial nitric oxide synthase (eNOS) [68]. Furthermore, SIRT1 suppresses the activation of the nucleotide-binding oligomerization domain-like receptor protein 3 (NLRP3) inflammasome, which is a multiprotein complex that is essential for mediating immune responses to cellular stress due to promoting caspase-1, which in turn increases the secretion of pro-inflammatory cytokines, such as IL-1β and IL-18 [69,70].

Additionally, SIRT1 activates the PI3K/Akt pathway, thereby suppressing oxidative stress-induced and mitochondria-dependent apoptosis, and promoting cell proliferation [71,72]. Through stimulation of Akt/FOXO3a axis and Nrf2 signaling, SIRT1 also mitigates ferroptosis, further showing its cytoprotective potential [73,74]. SIRT1-mediated upregulation of the FOXO family enhances the cellular response against oxidative stress by promoting the expression of antioxidant enzymes. Additionally, SIRT1 contributes to the activation of Nrf2, either directly or indirectly through the peroxisome proliferator-activated receptor-gamma coactivator-1 alpha/estrogen-related receptor alpha (PGC-1α/ERRα) pathway, thereby reinforcing the cellular antioxidant capacity [61,75,76].

Mitochondrial biogenesis enhances mitochondrial number as an adaptive response to increased energy demands. This process is primarily regulated by PGC-1α, which can be directly deacetylated by SIRT1 to activate nuclear transcription factors, including Nrf2 and FOXO3 [77,78,79]. SIRT1 also takes part in the regulation of mitochondria dynamics. Its activation leads to a significant decrease in the level of Drp1 and its translocation [80], thus preventing mitochondria fission and protecting mitochondria from further damage [81]. Additionally, SIRT1 deacetylates and activates c-Myc [82], and downregulation of FOXO1 and upregulation of c-Myc are directly linked to the Warburg effect [7], which suggests a correlation between SIRT1 and the Warburg effect, although it has not been described in the literature yet.

To investigate the complex mechanism of SIRT1 activity, various ligands and modulators have been employed (Table 1) [83]. SIRT1 expression can be regulated by synthetic molecules such as SRT1720 and SRT2104 [83], as well as by compounds naturally found in food [84,85], like quercetin, resveratrol, or fisetin (Figure 1) [86,87,88,89,90,91,92]. SIRT1 can be activated by compounds that increase NAD^+^ levels, including natural molecules such as resveratrol, quercetin, and berberine [93,94,95], as well as synthetic or endogenous regulators like amifostine [96], nicotinamide phosphoribosyltransferase (NAMPT) [97], and AMPK [98]. Resveratrol is the most commonly studied natural activator of SIRT1 with many downstream targets [99]; however, synthetic compounds exhibit a thousand-fold greater potency (Table 1) [100,101]. Unfortunately, SIRT1 activators are not fully selective (Table 1) and may also activate sirtuin 2 and sirtuin 3 (SIRT2 and SIRT3), although with a much lower potency [100,102]. SIRT2 predominantly locates in cytosol, whereas SIRT3 is a major deacetylase of mitochondria and both are distributed in metabolically active tissues, such as the brain, heart, skeletal muscle, liver and brown adipose tissue [103,104]. All of the above highlight the importance of careful selection of the modulator and modulator dose to provide the maximum therapeutic benefit and to avoid the off-target effects associated with activation of other sirtuins (SIRTs).

SIRT1 activators share some common effects associated with the above-mentioned functions, primarily antioxidative [7,105], anti-inflammatory [105,106], antiproliferative [7,16,63,106,107,108], hypotensive [16,63,105,106,107,108,109,110,111], and antihypertrophic [16,63,105,106,107,108,109,110,111,112] properties. Due to these properties, particularly the antioxidative and anti-inflammatory properties, SIRT1 activators may be useful as a complement to standard therapies, helping to alleviate symptoms and slow down disease progression.

**Table 1:** Potency of chosen sirtuin 1 (SIRT1) modulators.

Modulators		Potency	References
resveratrol– **activator**	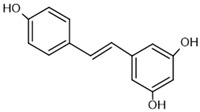	SIRT1: EC_50_ = 100 μM *EC_1.5_ = 31.6 μM SIRT2: EC_50_ = 1.92 μM *EC_1.5_ > 300 μM SIRT3: *EC_1.5_ > 300 μM	[100,102]
SRT1720– **activator**	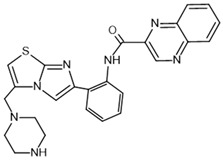	SIRT1: EC_50_ = 0.10 μM *EC_1.5_ = 0.16 μM SIRT2: *EC_1.5_ = 37 μM SIRT3: *EC_1.5_ > 300 μM	[100,102]
SRT2104– **activator**	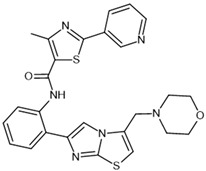	SIRT1: *EC_1.5_ = 450 nM	[113]
SRT1460– **activator**	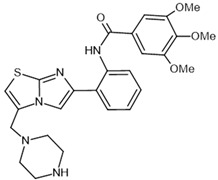	SIRT1: *EC_1.5_ = 2.9 μM SIRT2: *EC_1.5_ > 300 μM SIRT3: *EC_1.5_ > 300 μM	[102]
SRT2183– **activator**	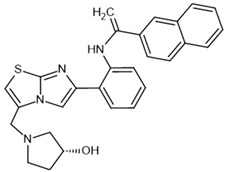	SIRT1: *EC_1.5_ = 0.36 μM	[102]
SRT3025– **activator**	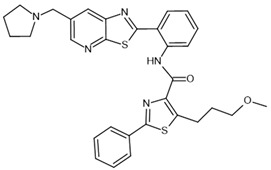	SIRT1: *EC_1.5_ < 1 µm	[114]
EX-527– **inhibitor**	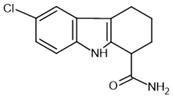	SIRT1: IC_50_ = 38 nM SIRT2: IC_50_ = 19.6 μM SIRT3: IC_50_ = 48.7 μM	[115]
nicotinamide– **inhibitor**	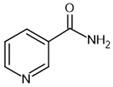	SIRT1: IC_50_ = 62 μM SIRT2: IC_50_ = 10 μM SIRT3: IC_50_ = 31 μM	[116]
sirtinol– **inhibitor**	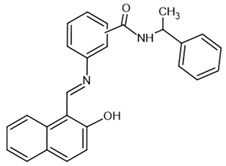	SIRT1: IC_50_ = 123.2 μM SIRT2: IC_50_ = 38 μM SIRT3: IC_50_: 189.0 μM	[117,118]
tenovin-6– **inhibitor**	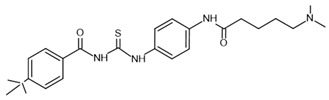	SIRT1: IC_50_ = 21 μM SIRT2: IC_50_ = 10 μM SIRT3: IC_50_ = 67 μM	[119]
suramin– **inhibitor**	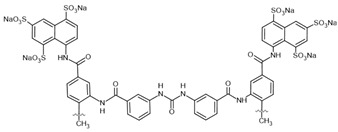	SIRT1: IC_50_ = 297 nM SIRT2: IC_50_ = 799 nM	[120]

*EC_1.5_—1.5-fold effective concentration, EC_50_—half-maximal effective concentration, IC_50_—half-maximal inhibitory concentration, SIRT1—sirtuin 1, SIRT2—sirtuin 2, SIRT3—sirtuin 3.

### Sirtuin 1 in the Cardiovascular System

SIRT1 acts in a multidirectional manner on the cardiovascular system, exerting protective effects [49,121,122,123], including systemic hypertension, atherosclerosis, vascular complications of diabetes, heart failure, myocardial infarction, ischemia and reperfusion injury, cardiomyopathy, cardiotoxicity, coronary heart disease, aortic aneurysm, or deep vein thrombosis [43,46,49,50,79,124,125,126,127,128,129,130,131,132], which was very elegantly reviewed by Ding et al. [58]. In fact, SIRT1 and its downstream pathways appear critical for both normal homeostasis and protection from cardiovascular disease (CVD)-induced defects [30]. In many CVDs the expression of SIRT1 was downregulated, such as hypertension, atherosclerosis, aortic aneurysm, hyperglycemia, and after ischemia/reperfusion (I/R) injury [58]. Moreover, significant developmental septal and valvular abnormalities in the hearts [133] or cardiac fibrosis and diastolic dysfunction [134] of mice lacking SIRT1 were observed.

**Figure 2:**
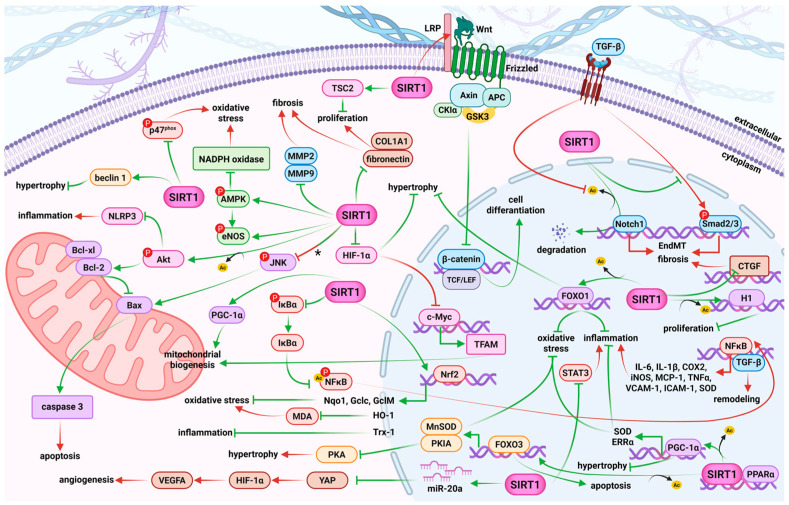
Signaling pathways activated by sirtuin 1 (SIRT1) activators in cardiovascular diseases including pulmonary (arterial) hypertension. Ac—acetylated/acetyl group, Akt—protein kinase B, AMPK—adenosine monophosphate-activated protein kinase, APC—adenomatous polyposis coli, Bax—Bcl-2-associated x-protein, Bcl-2—B-cell lymphoma 2, Bcl-xl—B-cell lymphoma-extra-large, c-Myc—cellular Myc, CKIα—casein kinase I alpha, COL1A1—collagen type I alpha I chain, COX2—cyclooxygenase 2, CTGF—connective tissue growth factor, EndMT—endothelial-to-mesenchymal transition, eNOS—endothelial nitric oxide synthase, ERRα—estrogen-related receptor alpha, FOXO1—forkhead box protein O1, FOXO3—forkhead box O3, Gclc—glutamate-cysteine ligase catalytic subunit, GclM—glutamate-cysteine ligase modifier subunit, GSK3—glycogen synthase kinase 3, H1—histone H1, HIF-1α—hypoxia-inducible factor-1 alpha, HO-1—heme oxygenase 1, ICAM-1—intercellular adhesion molecule 1, IL-1β—interleukin-1β, IL-6—interleukin-6, iNOS—inducible nitric oxide synthase, IκBα—NF-κB inhibitor alpha, JNK—Jun N-terminal kinase, LEF—lymphoid enhancer-binding factor, LRP—lipoprotein receptor-related protein, MCP-1—monocyte chemoattractant protein-1, MDA—malondialdehyde, miR-20a—microRNA-20a, MMP2—matrix metalloproteinase 2, MMP9—matrix metalloproteinase 9, MnSOD—manganese-dependent superoxide dismutase, NADPH—nicotinamide adenine dinucleotide phosphate, NF-κB—nuclear factor-kappa B, NLRP3—nucleotide-binding oligomerization domain-like receptor protein 3, Notch1—Neurogenic locus notch homolog protein 1, Nqo1—NADPH quinone oxidoreductase, Nrf2—nuclear factor erythroid 2-related factor 2, P—phosphorylated, p47phox—neutrophil cytosolic factor 1, PGC-1α—peroxisome proliferator-activated receptor-gamma coactivator-1 alpha, PKA—cAMP-dependent protein kinase, PKIA—cAMP-dependent protein kinase inhibitor, PPARα—peroxisome proliferator-activated receptor alpha, SIRT1—sirtuin 1, Smad2/3—mothers against decapentaplegic homolog 2/3, SOD—superoxide dismutase, STAT3—signal transducer and activator of transcription 3, TCF—T-cell factor, TFAM—mitochondrial transcription factor A, TGF-β—transforming growth factor-β, TNFα—tumor necrosis factor alpha, Trx-1—thioredoxin-1, TSC2—tuberous sclerosis complex subunit 2, VCAM-1—vascular cell adhesion molecule 1, VEGFA—vascular endothelial growth factor A, Wnt—wingless-type mouse mammary tumor virus integration site family, YAP—yes-associated protein 1. * Note that in the context of hypertension, heart failure, and cardiomyopathy, apoptosis may have a negative effect. In hypertension or pulmonary (arterial) hypertension, endothelial cell apoptosis may affect vascular function, leading to vascular dysfunction and accelerating the development of cardiovascular disease. However, in pulmonary (arterial) hypertension, in the case of hyperproliferating PASMCs it may be beneficial. Created in BioRender. Krzyżewska, A. (2025) https://BioRender.com/bs786aq (accessed on 21 August 2025).

SIRT1 regulates important metabolic and physiological processes, including stress resistance, metabolism, apoptosis, and energy balance [32,33,41]. It also reverses cholesterol transport and reduces the risk for development of atherosclerosis and CVD [42,58]. The pleiotropic function of SIRT1 in the cardiovascular system results from its regulation of a wide range of signaling pathways and molecular axes (Figure 2). Thus, SIRT1 is a multifunctional protective molecule in ECs by promoting endothelial angiogenesis and migration, improving endothelium-dependent vasodilatation, maintaining endothelial metabolism, preventing endothelial senescence, and suppressing vascular inflammation and oxidative stress [58,122,135]. It has the potential to reverse endothelial dysfunction related to obesity and aging in ex vivo human vessels [44,47], to regulate the proliferation and migration of endothelial progenitor cells, and to promote their differentiation via the wingless-type mouse mammary tumor virus integration site family/β-catenin/glycogen synthase kinase 3β (Wnt/β-catenin/GSK3β) signaling pathway in atherosclerotic mice [74].

In VSMCs, age-related SIRT1 reduction is linked with vascular senescence and inflammation and formation of abdominal aortic aneurysms, while SIRT1 overexpression provides a therapeutic target by diminishing NF-κB binding on the promoter of MCP-1 and blocking vascular inflammation [48]. In aortas of Ang II-induced hypertensive mice, the activity of SIRT1 was reduced since overexpression of SIRT1 or its activation by resveratrol reversed hypertension and vascular remodeling by reducing the binding of NF-κB on the promoter of TGF-β1 [47]. Similarly, in humans and rodents, SIRT1 has long been determined as an antiatherosclerosis factor by increased expression of eNOS, reduction in macrophage foam cell formation, and the uptake of oxidized low-density lipoproteins, lower levels of cholesterol, foam cells, and atherosclerotic plaque [58]. In diabetic mice SIRT1 exerts an antiangiogenic role in diabetic retinopathy via microRNA (miR)-20a elevation and yes-associated protein/HIF-1α/vascular endothelial growth factor A (YAP/HIF-1α/VEGFA) downregulation [136]. Moreover, SIRT1 modulates transcription factors such as NF-κB, HIF-α, and FOXO1 to reduce the severity of diabetic retinopathy [137].

SIRT1 also regulates cardiomyocyte hypertrophy, PANoptosis, endothelial-to-mesenchymal transition (EndMT), and the activation of cardiac fibroblasts [58]. Mechanistically, SIRT1, through interaction with peroxisome proliferator-activated receptor alpha (PPARα) via deacetylation of PGC-1α or FOXO3, reduces hypertrophy and oxidative stress in cardiomyocytes, respectively. Furthermore, SIRT1 mitigates cardiac hypertrophy via the FOXO3/cyclic adenosine monophosphate (cAMP)-dependent protein kinase inhibitor (FOXO3/PKIA) axis and through modulation of brain natriuretic peptide (BNP), atrial natriuretic peptide (ANP), HIF-1α, FOXO1, and Beclin 1 [132,137,138,139]. Activation of SIRT1 plays a crucial role in mitigating EndMT by modulating the neurogenic locus notch homolog protein 1 (Notch1) and TGF-β/Smad2/3 pathways, thereby reducing fibrotic responses associated with heart failure [134]. SIRT1 modulates the TGF-β signaling pathway through its interaction with Smad3, a canonical downstream effector. Upon TGF-β stimulation, Smad3 becomes acetylated, which promotes its transcriptional activity. Activation of SIRT1 leads to deacetylation of Smad3, thereby reducing its activity and attenuating TGF-β-induced signaling and fibrosis. Importantly, this effect requires catalytically active SIRT1, as the presence of an inactive SIRT1 mutant abolishes the ability to suppress Smad3 activity [140]. SIRT1 also effectively downregulates matrix metalloproteinase 2 (MMP2), MMP9 [141], ANP [142], collagen type I alpha I chain (COL1A1) [142,143,144], and connective tissue growth factor (CTGF) [142], key mediators of tissue remodeling and fibrosis in the heart and vasculature. In addition, SIRT1 regulates apoptosis by deacetylating FOXO3a in the heart tissue [127]. It also suppresses phosphorylated Jun N-terminal kinase (p-JNK) activation [145], thus leading to the inhibition of mitochondrial apoptotic pathways (B-cell lymphoma-2 (Bcl-2), Bcl-2-associated x-protein (Bax) B-cell lymphoma-extra-large (Bcl-xl), and caspase 3) in the heart of spontaneously hypertensive rats and rodents with myocardial infarction (MI), and in human umbilical vein endothelial cells (HUVECs) subjected to an oxidized low-density lipoprotein-induced model of deep vein thrombosis [127,144,145].

Similarly, SIRT1 also protects the heart against oxidative stress and inflammation by modulating NLRP3 inflammasome activation through the Akt-dependent metabolic regulation during ischemic injury [146]. Its role in protecting against oxidative stress involves activation of the Nrf2/heme oxygenase 1 (HO-1) and FOXO3a/manganese-dependent superoxide dismutase (FOXO3a/MnSOD) pathways, as well as modulation of FOXO1, phosphorylated neutrophil cytosolic factor 1 (p47phox), AMPK, NOX, PGC-1α, SOD, and malondialdehyde (MDA) levels. It also upregulates Nrf2 downstream targets, including NAD(P)H: quinone oxidoreductase-1 (Nqo1), glutamate-cysteine ligase catalytic subunit (Gclc), and glutamate-cysteine ligase modifier subunit (Gclm) [50,79,132,141,143,144,147,148,149,150]. The anti-inflammatory effects of SIRT1 activators are among the most thoroughly studied. They involve the modulation of inflammatory-related mediators such as TNF-α, MCP-1, interleukins, and SOD [141]. SIRT1 also exerts anti-inflammatory effects through FOXO1 deacetylation and inhibition of NF-κB inhibitor alpha (IκBα) phosphorylation and signal transducer and activator of transcription 3 (STAT3) [141,149].

Moreover, in animal models of heart failure with preserved ejection fraction (HFpEF), SIRT1 inhibits cardiac fibrosis via the Smad3 pathway [53]. In studies involving patients with HFpEF, an exercise training and caloric restriction program resulted in a hypotensive response and improved ejection fraction, which is partly attributable to significant increases in NAD^+^ levels and SIRT1 activity [151].

Sexual dimorphism in the cardiovascular system highlights the differing roles of SIRTs in CVD between genders. While much research has focused on male models, the significance of SIRTs in females, particularly in pregnancy-related CVD like preeclampsia, is increasingly recognized. Reduced SIRT1 expression in preeclamptic placentas and plasma correlates with symptoms such as hypertension and proteinuria, which can be reversed by SIRT1 supplementation or agonist treatment, underscoring the importance of considering sex differences in the cardiovascular research and potential therapies [58,152]. Interestingly, evidence from experimental studies indicates that estrogen, through the activation of SIRT1, has a protective effect on arteries, delaying their aging and the development of atherosclerosis [153]. Estradiol has been shown to increase the expression and activity of SIRT1 in numerous tissues, including the heart and blood vessels, while the absence of estradiol reduced SIRT1 activity, which was associated with metabolic disorders and promoted age-related diseases [154]. Another study showed that exposure to 17-β-estradiol alleviated the symptoms of postmenopausal metabolic syndrome in rats by improving vascular endothelial function and reducing cardiac apoptosis, which was associated with modulation of SIRT1/AMPK/H3 signaling [155]. Emerging reports highlight the need for further research.

In addition to preclinical studies, several ongoing clinical trials are evaluating the safety and efficacy of resveratrol in multiple CVDs, including peripheral arterial disease, coronary artery disease, hypertension, and heart failure. For instance, resveratrol supplementation has been shown to increase circulating SIRT1 levels, reduce left atrial remodeling, enhance left ventricular (LV) diastolic function, and mitigate cardiac fibrosis in patients (identifier: ChiCTR1800016122 and Unique identifier: NCT01668836 (reviewed by [58])).

Notably, in the few original works, including a recent comprehensive review on the roles of SIRTs in CVD [58], there was no mention of SIRT1 in the context of PAH, despite the extensive exploration of related topics. Understanding these aspects will be crucial for advancing research and therapeutic strategies in PAH and beyond. This review aims to provide a comprehensive overview of the mechanisms by which SIRT1 modulates the functions of cardiovascular cells in PAH/PH. We will explore therapeutic approaches that target SIRT1 for the prevention and treatment of PAH, addressing the remaining questions at the molecular, cellular, systemic, and clinical application levels. Given the ongoing development of SIRT activators and the extensive investigation of their biological functions in cardiovascular biology and diseases over the past 25 years, there is an urgent need for a discussion on the advances and challenges in this field in the context of PAH/PH.

## 4. Sirtuin 1 in Pulmonary (Arterial) Hypertension

There is a lack of data regarding the role of SIRT1 in PAH in humans; therefore, we primarily focused on experimental models. In this study, we reviewed the available literature on the role of SIRT1 in experimental PH, focusing on three primary models: (1) monocrotaline (MCT)-induced PH mainly in male Sprague-Dawley (SD) rats [106,110,111,112,156,157], and (2) hypoxia-induced PH, primarily in male SD rats [7,63,109,110,158], with one study conducted on Wistar rats [108] and one on SIRT1 knockout female mice [7]. Additionally, we examined (3) the Sugen–hypoxia model, which included experiments carried out in male SD rats [16] and a mouse model encompassing male or both sexes [16,152]. Given the limited availability of patient-derived cells, our in vitro analyses predominantly focused on healthy human PASMCs [7,159], while also incorporating cells from iPAH patients [7,16] and animal models subjected to disease induction [63,105,108] or hypoxic conditions [63,107,108,109,157,158,160,161], thus providing a representative framework for studying SIRT1-targeted interventions in PH/PAH.

### 4.1. Monocrotaline-Induced Pulmonary Hypertension

Monocrotaline, a pyrrolizidine alkaloid, induces PH in rats [162] with a single subcutaneous injection by mimicking features of human PAH, including endothelial cell apoptosis and oxidative stress and causes death in animals at 4–6 weeks after insult [106]. MCT disrupts eNOS and NO signaling, intracellular membrane trafficking, and over-proliferation of PASMCs, leading to PA medial hypertrophy and obstructive vascular remodeling. Rats with MCT-induced PH exhibit increased Fulton index (RV + septum (S) ratio/LV), right ventricular systolic pressure (RVSP), and mPAP, compared to the control group [163]. The MCT-injected rats exhibit increased lung congestion compared to control Wistar rats [164].

### 4.2. Hypoxia- and Sugen/Hypoxia-Induced Pulmonary Hypertension

Rodents are typically subjected to chronic hypoxia for 3–4 weeks to induce PH. Hypoxic exposure in rats results in vascular remodeling, endothelial cell dysfunction, and apoptosis in both the small PA and veins. Similarly, mice and rats exposed to chronic hypoxia exhibit pulmonary vascular remodeling and a rise in RVSP [165]. The hallmark of chronic hypoxia-induced pulmonary vascular remodeling is the muscularization of arterioles that are typically non-muscularized under physiological conditions [165]. Importantly, many of the hypoxia-induced alterations are reversible upon return to normoxic conditions, making the Hx-induced PH model particularly valuable for studying milder or early-stage forms of PH [165]. Sugen 5416 (SU5416) is an antagonist of vascular endothelial growth factor receptor 2 (VEGFR-2) that selectively binds to ECs, accumulates in the cell membrane, and is gradually released into the cytoplasm, allowing for sustained inhibition of VEGFR signaling [165]. A widely adopted experimental protocol entails administering a single subcutaneous dose of 20 mg/kg SU5416 to 6- to 8-week-old rats, followed by exposure to hypoxia (10% O_2_) for 3 weeks, and subsequently to normoxia from 1 to 10 weeks. A single administration of SU5416 combined with chronic hypoxia in rats induces PA medial hypertrophy, thickening of the PA wall, and persistent vasoconstriction, culminating in elevated PAP and the development of plexiform lesions. SU5416-mediated VEGF inhibition initially triggers apoptosis of PAECs, which is followed by the proliferation of both PAECs and PASMCs, along with the emergence of apoptosis-resistant PAECs. The dual-hit SU5416/hypoxia model results in more severe pulmonary hemodynamic impairment compared to either SU5416 or hypoxia alone [166].

### 4.3. Sirtuin 1 Expression in Pulmonary (Arterial) Hypertension

The role of SIRTs in PAH, their expression levels, and the lysine acetylation level in human PAH and experimental PH models have not yet been revealed in detail. Several publications have shown that SIRTs might exert a protective capacity by improving PH and right ventricle hypertrophy (RVH), especially SIRT1, SIRT3, and sirtuin 7 (SIRT7) in animal models [58].

Recent studies have highlighted the role of SIRTs in the pathophysiology of PH [58], with only a limited number investigating the expression of SIRT1 in different models related to PH (Table 2), specifically focusing on PASMCs and PAECs (Table 3). SIRT1 expression was downregulated in PH models in lungs [7,63,106,111,157,167], PA [110], PASMCs [7,16,63,105,108,109,156,157,159,160,161,167,168], and with tendency in the RV [45]; surprisingly, it was not altered in PASMCs from humans with PAH, but pharmacological and genetic inhibitions of SIRT1 promoted cell proliferation via the alteration of the acetylation/deacetylation balance [7]. Interestingly, SIRT1 expression was increased in rodent PAECs of the microvascular system, or in PASMCs under hypoxic conditions to promote cell proliferation and inhibit cell apoptosis [107,160], respectively. The modulation of SIRT1 expression by its various activators has been shown to significantly impact the protein’s levels. In general, various activators or adenoviral overexpression of SIRT1 caused an increase in its expression (Table 2 and Table 3). Only one study revealed no changes in SIRT1 expression after hypoxia exposure in Wistar rats after resveratrol treatment, despite its ability to decrease RVSP [108]. Similarly, there was no significant change, but only the tendency in overexpression of SIRT1 in the RV of SD rats with MCT-induced PH [112]. Nevertheless, there was a significant decrease in acetyl-Lysine (Ac-Lys) in the RV of SD rats, which can suggest that resveratrol could change only the activity of SIRT1 [112]. This regulatory effect is relatively independent of the PH model employed, the severity of the condition, the tissue type examined, and the genetic background of the animal strains utilized (Table 2 and Table 3).

Although the role of SIRT1 in PH remains controversial, especially regarding its expression under hypoxic conditions and its impact on proliferation and apoptosis, some studies suggest it may exert protective effects. Additionally, SIRT3 and SIRT7 are consistently downregulated in PH models, with their restoration shown to alleviate disease features. These findings highlight the importance of SIRTs in PH pathogenesis and therapy [58]. This review explores the therapeutic potential of targeting SIRT1 pathways, emphasizing the need for further research to clarify their roles.

### 4.4. Implications of Sirtuin 1 in the Pathogenesis of Pulmonary (Arterial) Hypertension

**Figure 3:**
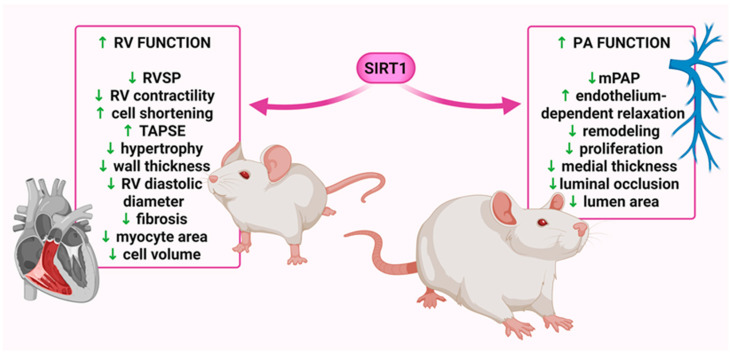
Effects of sirtuin 1 (SIRT1) activators in animal models of pulmonary hypertension. ↑—increase, ↓—decrease, mPAP—mean pulmonary arterial pressure, PA—pulmonary arteries, RV—right ventricle, RVSP—right ventricular systolic pressure, TAPSE—tricuspid annular plane systolic excursion. Created in BioRender. Krzyżewska, A. (2025) https://BioRender.com/s6o3a61 (accessed on 21 August 2025).

Various SIRT1 activators have been shown to modulate key pathophysiological features of PH, including RVSP, RV hypertrophy, vascular remodeling, inflammation, and oxidative stress (Table 2). Activators such as resveratrol [108,111,112,156,159,167], SRT1720 [108], SRT2104 [16,167], secreted Klotho (SKL) [106], adeno-associated overexpression of SIRT1 [110], short interfering RNA targeting Jagged2 (si-Jag2) [109], circular RNA derived from the SIRT1 gene (circ-SIRT1) [63], phoenixin-20 [158], scutellarein [157], hResistin [167], and CR [110] exert protective effects by reducing RVSP and/or mPAP [16,63,105,106,108,109,110,111,156,157,158,159], as well as RV hypertrophy (Fulton index, RV wall thickness) [16,63,105,106,108,109,110,111,112,156,157,158,159] and mitigating PA remodeling [7,16,106,108,109,110,111,156,157,158,159]. Beneficial effects of SIRT1 activators/modulators on PH have been demonstrated in both preventive [7,63,108,109,110,157,158] and therapeutic [16,105,106,111,112,156,159,167] experimental models, highlighting their potential translational relevance. Moreover, these effects have been observed in both MCT-treated [106,110,111,112,156,157] and hypoxia-induced [7,16,63,105,108,109,157,158,167] PH models in rats and mice (Figure 3).

Notably, resveratrol decreases hypertrophy markers, such as BNP and troponin C (TnC), and slows the progression of RV fibrosis [112], which is an irreversible change during PAH development and a leading cause of mortality among patients with this disease [13]. Moreover, it reduced cardiomyocyte area and inhibited the progression of fibrosis in RV, improved cell shortening, increased tricuspid annular plane systolic excursion (TAPSE), and prevented systolic failure, but had a limited effect on the development of MCT-induced PH changes in the vascular architecture of the echocardiographic PA hemodynamic, i.e., PA acceleration time (PAAT), PAAT/ejection time (ET) susceptible to changes in pulmonary vascular resistance, and impedance, and it shortens in correlation with an increase in systolic PAP and mPAP. The LV echocardiography findings did not show any change associated with increased mPAP (Figure 3) [112]. A minimal anti-remodeling effect, similar to that observed in the lungs, was also seen in the heart wall structure [169]. Using a higher dose and improving the administration route (i.e., using nebulization therapy) could be an effective way of improving these results [112].

**Figure 4:**
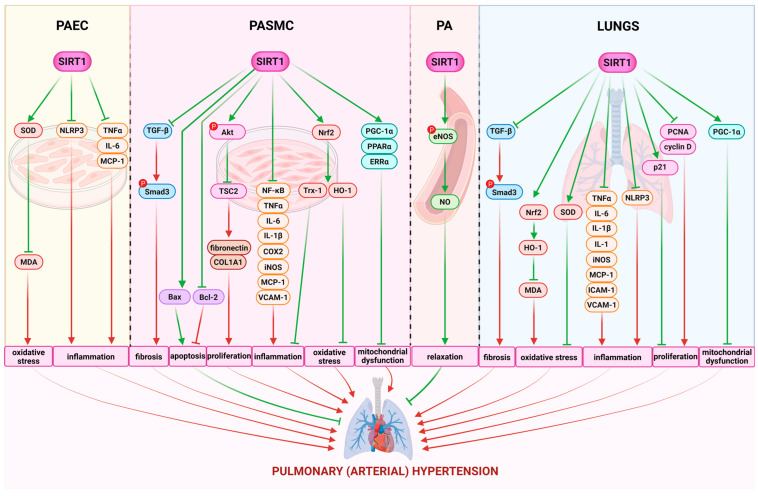
Molecular role of sirtuin 1 (SIRT1) activation in pulmonary (arterial) hypertension. Akt—protein kinase B, Bax—Bcl-2-associated x-protein, Bcl2—B-cell lymphoma-2, COL1A1—collagen type I alpha I chain, COX2—cyclooxygenase 2, eNOS—endothelial nitric oxide synthase, ERRα—estrogen-related receptor alpha, HO-1—heme oxygenase 1, ICAM-1—intercellular adhesion molecule 1, IL-1—interleukin 1, IL-1β—interleukin-1 β, IL-6—interleukin 6, iNOS—inducible nitic oxide synthase, MCP-1—monocyte chemotactic protein 1, MDA—malondialdehyde, NF-κB—nuclear factor-kappa B, NLRP3—nucleotide-binding oligomerization domain-like receptor protein 3, NO—nitric oxide, Nrf2—nuclear factor erythroid 2-related factor 2, PA—pulmonary arteries, PAECs—pulmonary artery endothelial cells, PASMCs—pulmonary artery smooth muscle cells, PCNA—proliferating cell nuclear antigen, PGC-1α—peroxisome proliferator-activated receptor-gamma coactivator-1 alpha, PPARα—peroxisome proliferator-activated receptor alpha, SIRT1—sirtuin 1, Smad3—mothers against decapentaplegic homolog 3, SOD—superoxide dismutase, TGF-β—tissue growth factor-β, TNFα—tumor necrosis alpha, Trx-1—thioredoxin-1, TSC2—tuberous sclerosis complex subunit 2 VCAM-1—vascular cell adhesion molecule 1. Created in BioRender. Krzyżewska, A. (2025) https://BioRender.com/n77dak2 (accessed on 21 August 2025).

SIRT1 is found in close proximity to and interacts with eNOS in the perinuclear cytoplasm of ECs [135]. By deacetylating eNOS, SIRT1 boosts its enzymatic activity, leading to increased NO synthesis, which is essential for preserving proper vascular function [110]. SIRT1 expression was downregulated in PA of MCT-induced PH rats, which in turn leads to diminished eNOS activity and lower NO production. This impairment contributed to endothelial dysfunction and the progression of PH [110,156]. Other studies demonstrated elevated levels of phosphorylated eNOS (p-eNOS) in the PA of MCT-induced PH rats following transfection with adenoviral vectors for SIRT1 overexpression and after a CR diet [110]. Additionally, increased p-eNOS levels were observed in the lungs of these rats after injection of mesenchymal stem cells (MSCs) overexpressing SKL [106]. However, short-term CR was shown to improve endothelial function, as demonstrated by a significant improvement of endothelium- and concentration-dependent vasorelaxation in response to acetylcholine (Ach), which was absent in the presence of N-nitro-L-arginine methyl ester (L-NAME). It highlights potential non-pharmacological intervention against PAH via SIRT1/eNOS pathways (Figure 2) [110]. Moreover, adenoviral SIRT1 overexpression [110] or resveratrol [156,159] exhibit similar endothelioprotective effects on PA in rats under CR with increased eNOS phosphorylation [110,156] or without changes in eNOS expression [110,159], which provides a new potential therapeutic target in the development of PH (Figure 4).

PASMCs are the primary components of pulmonary vessels, and their proliferation and resistance to apoptosis contribute to lumen stenosis and wall stiffness, facilitating the occurrence and progression of PH. In PAH, alongside vascular endothelial dysfunction, several abnormal phenotypes of PASMCs are observed, including (1) enhanced proliferation and increased migration, (2) resistance to apoptosis, (3) oxidative stress, (4) mitochondrial dysregulation, and (5) heightened inflammatory responses (Figure 4) [7,58,105].

To further elucidate the mechanisms underlying SIRT1-dependent effects, we investigated its role with a focus on the hyperproliferation of PASMCs. In some studies, it has been shown that downregulation or inhibition of SIRT1 stimulates the proliferation and migration of PASMCs [7,63], whereas activators or overexpression of SIRT1 inhibit PASMC proliferation [7,16,63,107,108,109,156,157]. Specifically, in human PAH PASMCs, treatment with the SIRT1 activator SRT2104 significantly increases the expression of TSC2 (Figure 2), which downregulates proliferation via inhibition of mTORC1. This treatment also decreases the phosphorylation of Ser473 in Akt, reduces the abundance of collagen 1A (Col1A) production, and promotes apoptosis. Additionally, SRT2104 reduces the proliferation of control PASMCs on stiff matrices [16]. Arresting cells in the gap 0/gap 1 (G0/G1) phase of the cell cycle is essential for the development of vascular remodeling. Cyclin-dependent kinase inhibitor 1A (p21), cyclin D1, and cyclin E are crucial regulators of VSMC proliferation. SIRT1 overexpression counteracts the effects of PDGF-BB on cell cycle regulators by increasing p21 expression levels and decreasing the expression of cyclin D1, cyclin E, and cyclin-dependent kinase 2 (CDK2). This results in the accumulation of human PASMCs in the G0/G1 phase, thereby inhibiting vascular remodeling [111]. Furthermore, Circ-SIRT1 has been shown to reduce the proliferation and migration of PASMCs in rats, as evidenced by significant decreases in Smad3, mothers against decapentaplegic homolog 7 (Smad7), and TGF-β1 levels in the lungs of rats with hypoxia-induced PH. Additionally, PASMC markers such as VCAM-1 and alpha smooth muscle actin (α-SMA) were downregulated, along with a reduction in proliferating cell nuclear antigen (PCNA) levels, suggesting alleviation of PH progression [63]. Specifically, scutellarein effectively reduced hypoxia-induced Akt1, proto-oncogene, non-receptor tyrosine kinase Src (SRC), epidermal growth factor receptor (EGFR), MMP9, and prostaglandin-endoperoxide synthase 2 (PTGS2) expression in human PAH PASMCs in a SIRT1-dependent manner [157]. Potential SIRT1-mediated antiproliferative mechanisms have also been suggested, such as the normalization of BMP-4 and Smad signaling pathway components (Smad1/4) in the lung [156] and an increase in atrogin-1 levels [159].

Hypoxia triggers mitochondrial damage, leading to oxidative stress, inflammation, and pulmonary vascular remodeling. Mitochondrial dysfunction in PH is characterized by decreased mitochondrial biogenesis, altered mitochondrial dynamics, and the accumulation of mitochondrial ROS. SIRT1 plays an essential role in the regulation of mitochondrial function by deacetylating numerous mitochondrial proteins, including components of the electron transport chain and mitochondrial biogenesis regulator. Jag2 inhibition not only inhibited PASMC proliferation but also restored hypoxia-induced oxidative stress injury and mitochondrial dysfunction [109]. In PAH, the master regulator of mitochondrial biogenesis, PGC-1α, and its downstream targets (SIRT1, TFAM, and AMPK) are diminished in PASMCs. Zurlo et al. [7] revealed that human PAH PASMCs exhibit an altered acetylated/deacetylated state, characterized by increased acetylation of SIRT1 targets, including histone H1 and FOXO1. Activation of SIRT1 with the compound STAC-3 reduced histone H1 and FOXO1 acetylation, effectively inhibiting rat PASMC proliferation without affecting cell viability, and promoting mitochondrial biogenesis, as evidenced by elevated mitochondrial markers and PGC-1α targets. The reduced expression of mitochondrial mass markers, voltage dependent anion channel (VDAC) and citrate synthase, in PAH PASMC indicates impaired mitochondrial biogenesis. STAC-3 increases the expression of different factors maintaining mitochondrial biogenesis (PPARα, ERRα, Nrf2, TFAM) and prevents PDGF-induced mitochondrial fragmentation [7]. SIRT1, via deacetylation of an enzyme located in the inner membrane of mitochondria nicotinamide nucleotide transhydrogenase (NNT), restores mitochondrial NAD^+^/NADPH balance, regulates mitochondrial homeostasis, and counteracts the migration and proliferation of PASMCs induced by hypoxia [157]. Moreover, SIRT1 activation increased PASMC apoptosis by inducing mitochondrial permeability transition (mPT) dysfunction and led to dysfunction in the mitochondria by inducing nuclear pyknosis and mitochondrial swelling [108]. Therefore, targeting SIRT1 activity through the restoration of mitochondrial biogenesis and normalization of the PASMC phenotype may represent a novel therapeutic approach to inhibit PASMC proliferation and delay the progression of PAH [8].

SOD, MPO, and MDA serve as markers of inflammation and oxidative stress [109]. Moreover, MPO exhibits profibrotic and vasoconstrictive effects and contributes to the reduction in NO bioavailability, thereby impairing vascular function in PH [170]. A study revealed that transfection with adeno-associated virus serotype 1-Jag2 significantly increased SOD activity and decreased activity of MPO and MDA in the lungs of SD rats with hypoxia-induced PH [109]. Similarly, phoenixin-20 repressed oxidative stress (upregulated SOD and downregulated MDA) in both lung tissues of PH rats and hypoxia-stimulated pulmonary microvascular endothelial cells (PMECs) [158]. In addition, an important pathway involved in the regulation of oxidative stress is the Nrf2/HO-1 pathway, along with another Nrf2 target gene, Trx-1 (Figure 2). Hypoxia exposure promotes a reduction in these antioxidants levels, resulting in the impaired balance between oxidation and antioxidation [109]. Nrf2, HO-1, and Trx-1 were elevated in the lungs of SD rats with hypoxia-induced PH after transfection with M1 macrophage exosomes with miR-663b low expression [105] and adeno-associated virus serotype 1-Jag2 [109]. Moreover, reduction in vascular oxidative stress by SIRT1 activators was associated with downregulation of NOX-1 expression and glycoprotein 91-phagocyte oxidase (gp91phox) [156].

Inflammation plays a significant role in PH pathogenesis. Widespread macrophage infiltration was observed by the IHC analysis surrounding the small PA (diameter 50–80 μm) and throughout the lung tissue, suggesting a strong inflammatory response in rats with MCT-induced PH. In the lung tissues of the PH rats, the inflammation—as indicated by the cluster of differentiation 68 (CD68) marker level—was increased [106], and the profiles of iNOS and COX2 were heightened [105]. MSCs overexpressing SKL could effectively decrease the inflammation as the CD68 marker level was diminished in the PA and lungs [106]. In addition, expression of inflammatory cytokine genes related to PAH, i.e., TNFα, [105,156,157,158], interleukin (IL) 1β, IL6 [105,156,157,158], iNOS, and COX2 [105], were decreased in the PA and lungs of rats with hypoxia-induced PH transfected with M1 macrophage exosomes with miR-663b [105], or treated with the SIRT1 activator phoenixin-20 in hypoxic rats [158] or scutellarein in MCT-induced PH rats and hypoxia-induced PH mice [157], which was found to alleviate PH progression (Figure 2). Moreover, NLRP3 is a receptor in innate immune cells activated in both PAH rats and hypoxia-stimulated PMECs; it was markedly abolished by administration of phoenixin-20, implying the involvement of NLRP3 inhibition in SIRT1 function against PH (Figure 2) [157,158]. Additionally, SIRT1 exerts an anti-inflammatory effect in PH by quenching the post-translational acetylation and activation of high-mobility group box 1 (HMGB1) in macrophages. Furthermore, the suppression of SIRT1 signaling in pulmonary macrophages during the early post-hypoxic period may contribute to the vascular remodeling observed in PH, highlighting its potential as a therapeutic target in this disease [167].

The potent anti-inflammatory effects of SIRT1, mediated through transcriptional and epigenetic mechanisms, suggest that modulation of SIRT1 activity could be exploited as a therapeutic approach to control vascular inflammation in PAH. SIRT1 negatively regulates inflammatory gene transcription through multiple mechanisms. SIRT1 directly deacetylates the NF-κB p65 subunit at lysine 310, thereby suppressing NF-κB transcriptional activity and downstream pro-inflammatory gene expression. This effect is further reinforced by the ability of SIRT1 to prevent IκB degradation and nuclear translocation of NF-κB, attenuating cytokine release in activated endothelial cells and macrophages [171,172]. Overexpression of SIRT1 enhances the interaction between PPARα and P65, inhibiting the activation of NF-κB, and thus suppressing transcription of the inflammatory cytokine MCP-1 [173]. In addition, SIRT1-mediated deacetylation of histones in the promoter region of target genes directly inhibits target gene transcription, a mechanism also relevant in PAH, where it can suppress TNF-α, IL-1β, and IL-6 transcription [174]. SIRT1 directly interacts with HIF-1α, mediating its deacetylation at Lys374 and preventing p300 recruitment, which inactivates HIF-1α and thereby suppresses hypoxia-induced expression of IL-6, IL-8, and TNF-α [175]. Taken together, these findings underscore that impaired SIRT1-dependent signaling contributes to the overexpression of inflammatory genes in PAH, while therapeutic strategies aimed at restoring SIRT1 activity may provide significant anti-inflammatory and vasoprotective benefits.

Therefore, targeting SIRT1 activity—through its anti-inflammatory, antioxidant, antiproliferative, and pro-apoptotic effects, as well as by restoring mitochondrial biogenesis, may lead to improvement of the PASMC phenotype and may represent a novel therapeutic approach to inhibit vascular remodeling and delay the progression of PAH (summarized in Figure 4).

## 5. Limitations

Despite growing interest in SIRT1 as a therapeutic target in PAH, current studies are constrained by several critical limitations. The usage of SIRT1 activators has some limitations despite their cell-protective properties. Alcendor et al. [176] discovered that while a high SIRT1 level (12.5-fold overexpression) had negative effects, a moderate overexpression (2.5- to 7.5-fold) had protective effects. In transgenic SIRT1 mice, lower SIRT1 levels encouraged antioxidative activity, whereas greater levels caused cardiomyopathy, possibly as a compensatory reaction to increased antioxidant levels [177].

The bioavailability and possible adverse effects of SIRT1 activators are further important considerations. Resveratrol, for instance, has an extremely low bioavailability (less than 1%) [178], although this can be enhanced by choosing the proper drug delivery method [179]. A study revealed that resveratrol inclusion complex showed markedly improved oral pharmacokinetics in humans, characterized by faster absorption, greater absorption efficiency, and enhanced relative bioavailability. Furthermore, chemical modification of resveratrol into prodrugs or derivatives improves its metabolic stability, membrane permeability, and bioavailability, with resveratrol formulation T1 showing markedly better absorption and overall pharmacokinetic performance than T2 [180]. The metabolism of resveratrol is influenced by gut microbiota, with certain species converting it into metabolites such as dihydroresveratrol that may enhance its pharmacological activity. Individual differences in microbiota composition, as well as physiological factors, including age, sex, and health status, contribute to variability in bioavailability and efficacy. Moreover, co-administration with fats or bio-enhancers (such as piperine) can improve absorption by modulating intestinal permeability or metabolic enzyme activity [180]. Notably, resveratrol may result in gastrointestinal problems at higher dosages [181]. In the recent clinical trial involving healthy subjects, 5 out of 12 participants (41.7%) experienced adverse events possibly related to the resveratrol, which improved or resolved upon follow-up [180]. It is also important to consider circadian variations in bioavailability of SIRT1 activators. Almeida et al. [182] observed that administering resveratrol in the morning improved its bioavailability. Furthermore, all these features must be thoroughly examined in the context of PAH for a proper evaluation of SIRT1 activators’ therapeutic potential. The small difference between the beneficial and adverse effects of SIRT1 activation points to the need for accurate dosing techniques. Understanding how to maximize drug availability and administration time is crucial for deciding on the most effective and safe approach to treatment.

Although resveratrol seems a promising compound, there is its analogue, pterostilbene, being researched. It is more stable, more active, and exerts higher bioavailability than resveratrol, due to more lipophilic structure [183]. Further study is required to identify the best SIRT1 activator, optimal activation level and application strategies for PAH patients. Moreover, many investigations rely on non-specific activators (e.g., resveratrol), lack rigorous in vivo validation, focus narrowly on individual signaling pathways, or are restricted to select cell types, limiting the translational relevance of their findings [7,107,108,156]. Mechanistic understanding remains fragmented, with incomplete delineation of downstream networks and limited exploration of crosstalk with other regulatory pathways [16,63,160]. Addressing this gap will be critical for a mechanistic understanding of how SIRT1 integrates diverse regulatory networks to confer vascular protection and alleviate PAH.

Preclinical models are often restricted to single rodent paradigms (monocrotaline- or hypoxia-induced), which insufficiently capture the heterogeneity and complexity of human PAH, e.g., [7,63,107,108,156,158,159,160,168].

Translational potential is further hampered by sparse data on long-term safety, systemic effects, and clinical relevance [105,106,110,111,112]. Methodological limitations, including short treatment durations, small cohorts, and emphasis on preventive rather than therapeutic interventions, reduce robustness [111,156,161]. Emerging studies on novel small molecules and RNA regulators remain preliminary, with limited characterization of their physiological roles, regulatory networks, and safety profiles [63,105,160,168].

Currently, the number of studies clearly linking SIRT1, estrogens, and PAH pathogenesis is very limited. Although available experimental cardiovascular research results indicate interactions between estrogen signaling and SIRT1 activity [153,154], Shen et al. [16] did not observe differences in response to SRT104 treatment between male and female mice exposed to SuHx. However, in the context of PAH, this is the only study that has directly compared SIRT1 activators between sexes; so it cannot be ruled out that the effect of SIRT1 activators is modulated by hormonal factors; this requires further verification in larger, prospective analyses as it may be an important and promising direction for future research.

Furthermore, the complex interplay between SIRT1 and obesity in patients with PAH warrants further investigation. In individuals with obesity, there is a reduction in SIRT1 expression [44] which leads to a loss of its protective functions, including antioxidative properties. Moreover, there is limited and conflicting data regarding the prevalence and consequences of obesity in the PAH population. Although obesity is reported to have a protective effect on mortality in PAH—a phenomenon known as the obesity paradox—others find no significant effect of obesity on mortality. Additionally, body mass index (BMI) does not necessarily correlate with fat mass, which may serve as a more accurate predictor of mortality [184]. Therefore, the reduced expression of SIRT1 in obese individuals could significantly impact the pathophysiology of PAH. Conversely, in non-obese individuals, SIRT1 may be more effective in maintaining its protective functions, suggesting that its antioxidative activity could contribute to improved vascular health. Additionally, genetic variation in SIRT1 might influence lung function and human longevity by modulating subclinical inflammation arising from abdominal adipose tissue, further underscoring its significance in the context of obesity and related diseases [185]. Therefore, personalizing PAH treatment in this context could be a valid approach; however, further preclinical and clinical research is essential to fully understand the implications of SIRT1 as a potential therapeutic target, independent of body mass.

Collectively, these gaps underscore the urgent need for multi-model validation, comprehensive evaluation across diverse cell types, development of selective SIRT1 modulators, and integrative preclinical-to-clinical strategies to accelerate translation.

## 6. Conclusions

In summary, the present review highlights the pivotal role of SIRT1 in the pathophysiology of PAH/PH. SIRT1 is known to be downregulated in the context of PH, leading to a disruption of its protective effects. The beneficial actions of SIRT1 activation extend to the PASMCs, PAECs, PA, lungs, and RV and have been demonstrated in both preventive and therapeutic experimental models, as illustrated in Figure 3 and Figure 4. This sirtuin demonstrates antiproliferative, pro-apoptotic, anti-inflammatory, antioxidant, anti-fibrotic, and vasorelaxant properties via diverse signaling pathways. Moreover, modulation of SIRT1 activity has been shown to restore mitochondrial biogenesis and normalize the phenotype of PASMCs, thereby offering a novel therapeutic approach to mitigate the hyperproliferation characteristic of PAH. Preclinical studies have shown that activators and overexpression of SIRT1 positively influence RV function both hemodynamically and morphologically, while also reducing pulmonary vascular resistance and mitigating remodeling of the pulmonary arteries. These mechanisms are crucial points of focus for the latest therapies in PAH. Therefore, it is posited that SIRT1 may serve as a valuable adjunctive therapy in the management of PAH.

Looking ahead, future research should prioritize several critical directions: (1) further mechanistic exploration into the precise molecular mechanisms, including interactions with various sirtuin isoforms and their roles in endothelial function and vascular remodeling. Despite these promising insights, there remains a significant gap in the availability of selective and more bioavailable pharmacological tools to further elucidate the unprecedented role of SIRT1 in this context. In addition, multi-model validation is needed to confirm findings across different disease mechanisms and comprehensive cell type analysis across PAECs, PASMCs, and adventitial cells. (2) There is an urgent need for large-scale clinical trials to ascertain the therapeutic efficacy and safety of SIRT1 modulators in patients with PAH, particularly in advanced disease stages, as these outcomes may be significantly influenced by sex and the presence of obesity, including long-term investigations. The current absence of human studies underscores the necessity of this area of investigation. (3) Synergistic strategies for SIRT1 activation in conjunction with other therapeutic modalities, including gene therapy and lifestyle interventions such as caloric restriction, should be explored. By addressing these research avenues, future investigations can facilitate the development of innovative therapeutic strategies aimed at reversing the pathological processes associated with PAH and ultimately enhancing patient outcomes.

## 7. Methods

To find the most relevant articles on the SIRT1 role in PH, PubMed and Web of Science (WoS) databases were searched. We did not restrict publication dates in order to include both foundational and recent studies.

The term “SIRT1” was used as the primary keyword, and the search was refined by adding the secondary terms “pulmonary” and “hypertension” with the Boolean operator AND, which yielded 82 results in total. Only full-text original articles were included. First, the titles were analyzed, then the abstracts and full texts of articles, and duplicated or ineligible papers were removed. Subsequently, we excluded non-relevant records based on titles and abstracts. These included studies that did not refer to PH or SIRT1, involved animal species other than rats or mice, and were conducted on the material not relevant to this review. Other exclusion criteria were language other than English and studies on other sirtuins. Additionally, one study was identified through manual screening of the reference list of an included article.

Finally, 18 publications were included in this review and are demonstrated in Table 2 and Table 3. Further background details were derived from additional reviews and original articles listed in the references.

For the figure preparation, the software used was BioRender and ChemSketch 2021.

## Figures and Tables

**Table 2:** Effects of sirtuin 1 (SIRT1) modulators on various models of pulmonary hypertension.

Rodents Specifications	Conditions/ Comments	Modulator	Administration	SIRT1 Expression in PH	Effects After Modulator					Ref.
Species/Age/ Weight/Sex			Dose/Concentration/ Route/Duration/Time		RVSP	PAP/ mPAP	RV Hypertrophy	PA Wall Thickness	Result/s	
**Preventive models**										
SIRT1 KO mice (mice C57BL/6J); 5–6 w; n/d; female	21 d in hypoxia (10% O_2_)	C57BL/6J carrying both the UBC-Cre-ERT2 and the SIRT1floxDE4/ floxDE4 alleles)	tamoxifene (30 mg/kg/d) for 4 consecutive d to induce SIRT1 deletion, 21 d before exposure to hypoxia	↓ in lungs	↑	n/d	↑	↓↓ non-muscular ↔ part. muscular ↑ tot. muscular	in lungs: ↑ α-SMA, acetylation; ↓ PPARα, PGC-1α, HIF-1α; ↑↑ GLUT1, mitochondrial biogenesis	[7]
SD rats; 6 w; 150–200 g; male	40 d in hypoxia (10% O_2_)	circ-SIRT1	1 × 10^8^ CFU; i.v.; once, at 1 and 20 d of 40 d treatment	↓ in PASMCs and lungs	↓	n/d	↓	↓ pulmonary small blood vessels thickness and lumen stenosis	↑ SIRT1 expression in PASMCs and lungs; in lungs: ↓ Smad3, Smad7, TGF-β1, VCAM-1, α-SMA, ICAM-1, PCNA, vimentin	[63]
Wistar rats; n/d; 200–300 g; male	21 d, fractional inspired oxygen of 0.21 and 0.12	resveratrol	25 mg/kg/d; n/d; 21 d	↓↓ in PASMCs	↓↓	n/d	↓	↓	↔ SIRT1 expression in PASMCs	[108]
		SRT1720	25 mg/kg/d; n/d; 21 d	↓ in PASMCs	↓	n/d	n/d	↓	↔ SIRT1 expression in PASMCs	
SD rats; 6–8 w; 180–240 g; male	28 d in hypoxia (10% O_2_)	adeno-associated virus serotype 1-Jag2	n/d; 14 d before exposure to hypoxia	n/d	↓↓	n/d	↓↓	↓↓↓ <50 μm, ↓↓ >50 μm	in lungs: ↑↑↑ SOD; ↓↓ MPO, MDA activity; ↑↑ Nrf2, HO-1	[109]
SD rats; n/d; 240–260 g, male	60 mg/kg of MCT, 21 d (PAP, PA relaxation) or 7 d (eNOS level, SIRT1 expression) before evaluation	adenoviral vectors for the overexpression of SIRT1	7.5 × 10^9^ pfu; i.t.; 1 d before MCT injection	n/d	n/d	↓	n/d	n/d	↑↑ SIRT1 expression; in PA: ↑ p-eNOS; ↓↓ ac-eNOS	[110]
	60 mg/kg of MCT, 21 d before evaluation	short-term CR	10% restriction; 14 d before MCT injection +35% restriction; p.o.; 21 d after MCT injection	↓ in PA	n/d	↓	↓↓	↓	↑ SIRT1 expression; in PA: ↔ eNOS; ↑ p-eNOS; ↓↓ ac-eNOS	
SD rats; 6–8 w; 200–220 g; male	60 mg/kg of MCT, 21 d before evaluation	scutellarein	50 mg/kg/d; i.p.	↓↓ in lungs	↓↓	n/d	↓↓	↓↓	↑↑ SIRT1 expression; in serum: ↓↓ TNF-α, IL-6, IL-1β, α-SMA	[157]
C57BL/6 mice; 6–8 w; 20–22 g; male	28 d in hypoxia (10% O_2_) + SU5416 injection (20 mg/kg i.p.) once a week		10 mg/kg/d; i.p.	↓↓ in lungs	↓↓	n/d	↓↓	↓↓	↑↑ SIRT1 expression; in serum: ↓↓ TNF-α, IL-6, IL-1β, α-SMA	
n/d rats; 7–9 w; n/d; male	28 d in hypoxia (10.5% O_2_)	phoenixin-20	100 ng/g/d; 28 d; n/d	n/d	↓	↓	↓	↓	in lungs: ↓ TNF-α, IL-6, MCP-1, MDA, NLRP3, ASC; ↑ SOD activity	[158]
**Therapeutic models**										
SD rats; 6–8 w; n/d; male	21 d in hypoxia (10% O_2_) and 35 d in normoxia, SU5416 injection (20 mg/kg) on day 0 of the experiment	SRT2104	100 mg/kg/d, by gavage; at the beginning of w 4 of the experiment for 5 w, 5 d/w	n/d	↓	↓	↓↓↓	↓↓	n/d	[16]
C57BL/6J mice; n/d; n/d; female, male	35 d in hypoxia (10% O_2_), SU5416 injections (20 mg/kg) on d 0, 7 and 14 of the experiment		100 mg/kg/d, by gavage; at w 4, 5 d/w, 7 d from day 15 after PH induction	n/d	↓	↓	↓↓↓	↓	n/d	
SD rats; 6–8 w; 200–250 g; male	28 d in hypobaric conditions (pressure—380 mmHg, PaO_2_—79.6 mmHg)	exosomes derived from M1 macrophage with miR-663b low expression	20 μg of M1^miR-663b-in-^Exo; i.v.; 7 d, from d 30	↓↓↓↓ in PASMCs	↓	n/d	↓	n/d	↑ SIRT1 expression in PASMCs; in PASMCs: ↓ TNF-α, IL-6, IL-1β, iNOS, COX2; ↑ Nrf2, HO-1, Trx-1, AMPK	[105]
SD rats; 6–8 w; n/d; male	60 mg/kg of MCT, 21 d before evaluation	MSC overexpressing secreted KL	3.5 × 10^6^ MSC/rat; i.v.; once, 3 d after MCT injection	↓↓↓ in lungs	↓	n/d	↓	↓ PASMC proliferation; ↑ lumen area	↔ SIRT1 expression; in lungs: ↔ eNOS; ↑↑ p-eNOS; ↓ CD68	[106]
SD rats; adult; 280–300 g; male	60 mg/kg of MCT, 14 d before evaluation	resveratrol	2.5 mg/kg/d; p.o.; for 14 d after MCT injection or for 21 d after MCT injection	↓↓↓ in lungs	↓	↓	↔	↔ 25–50 μm; ↓↓ 51–100 μm; ↓ 101–500 μm	↑↑ SIRT1 expression in lungs; ↑↑ p21; ↔ cyclin D	[111]
	60 mg/kg of MCT, 21 d before evaluation				↓↓↓	↓↓↓	↓↓↓	↓↓ 25–50 μm; ↓↓ 51–100 μm; ↓ 101–500 μm	↑↑ SIRT1 expression in lungs; ↑↑ p21; ↓↓↓ cyclin D	
	60 mg/kg of MCT, 14 d before evaluation		20 mg/kg/d; p.o.; for 14 d after MCT injection or for 21 d after MCT injection		↓	↓↓↓	↓	↔ 25–50 μm; ↓↓ 51–100 μm; ↓↓↓ 101–500 μm	↑ SIRT1 expression in lungs; ↑↑↑ p21; ↓↓↓ cyclin D	
	60 mg/kg of MCT, 21 d before evaluation				↓↓↓	↓↓↓	↓↓↓	↓↓ 25–50 μm; ↓↓ 51–100 μm; ↓↓ 101–500 μm	↑↑ SIRT1 expression in lungs; ↑ p21; ↓↓↓ cyclin D	
SD rats; adult; >300 g; male	60 mg/kg of MCT	resveratrol	20 mg/kg/d; by gavage for 42 d after MCT injection	↔ in RV	n/d	↔	↓	n/d	in RV: ↓ BNP, TnC, Ac-Lys; ↔ Col1, IL-1β, IL-10	[112]
SD rats; adult; 300 g; male	60 mg/kg of MCT, 14 d before evaluation	resveratrol	25 mg/kg/d; p.o., in the drinking water; for 14 d after MCT injection or for 21 d after MCT injection	n/d	↓	n/d	↓	↓	n/d	[156]
	60 mg/kg of MCT, 21 d before evaluation			n/d	↓	n/d	↓	↓	in lungs: ↓ IL-6, IL-1, TNFα, PDGFα, PDGFβ, MCP-1, iNOS, ICAM-1, in PA: ↓ NOX-1; ↑ eNOS	
SD rats; 8–10 w; >300 g; male	50 mg/kg of MCT, 28 d before evaluation	resveratrol	3 mg/kg/d, p.o. in the drinking water; for 14 d from d 28 after PH induction	n/d	↓	n/d	↓	↔ <75 μm, ↓ 75–150 μm, ↔ >150 μm	in PA: ↑ atrogin-1; ↔ MuRF-1, eNOS, Kv1.5	[159]
SD rats; 4–5 w; 180–220 g; male	60 mg/kg of MCT	sh-circSIRT1	2 × 10^8^ TU/mL sh-RNA lentiviral vector of stably targeting circ-SIRT1; i.v.	n/d	n/d	↓	↓	↓	in PA: ↑ miR-145-5p	[168]

↑—increase, ↓—decrease, ↓/↑ *p* < 0.05, ↓↓/↑↑ *p* < 0.01, ↓↓↓/↑↑↑ *p* < 0.001, ↓↓↓↓ p < 0.0001, ↔ no change, ac-eNOS—acetylated endothelial nitric oxide synthase, Ac-Lys—acetylated lysine, AMPK—AMP-activated kinase, ASC—apoptosis-associated speck-like protein containing a CARD, BNP—brain natriuretic peptide, CD68—cluster of differentiation 68, CFU—colony-forming unit, circ-Sirt1—circular RNA SIRT1, Col1—collagen 1, COX2—cyclooxygenase 2, CR—calorie restriction, d—day/days, eNOS—endothelial nitric oxide synthase, evaluation—the assessment of treatment effects, GLUT1—glucose transporter 1, HO-1—heme oxygenase-1, HIF-1α—hypoxia-inducible factors alpha, i.v.—intravenous, ICAM-1—intercellular adhesion molecule 1, i.t.—intratracheally, IL-1—interleukin 1, IL-10—interleukin-10, IL-1β—interleukin-1β, IL-6—interleukin-6, iNOS—inducible nitric oxide synthase, KL—klotho, Kv1.5—voltage-gated potassium channel, M1^miR-663b-in^-Exo—M1 macrophage exosomes with miR-663b low expression, MCP-1—monocyte chemoattractant protein-1, MCT—monocrotaline, MDA—malondialdehyde, MPO—myeloperoxidase, MSC—mesenchymal stem cells, MuRF-1—muscle RING-finger protein-1, n/d—not determined, NLRP3—domains-containing protein 3, NOX-1—NADPH oxidase-1, Nrf2—nuclear factor erythroid 2-related factor 2, p.o.—per os, p21—cyclin-dependent kinase inhibitor 1A, PA—pulmonary artery, PAP—pulmonary arterial pressure, part.—partially, PASMCs—pulmonary arterial smooth muscle cells, PCNA—proliferating cell nuclear antigen, PDGFα—platelet-derived growth factor receptor alpha, PDGFβ—platelet-derived growth factor receptor beta, p-eNOS—phosphorylated endothelial nitric oxide synthase, pfu—plaque-forming units, PGC-1α—peroxisome proliferator-activated receptor gamma coactivator 1-alpha, PPARα—peroxisome proliferator-activated receptor alpha, Ref.—reference, RV—right ventricle, SD—Sprague-Dawley, serotype 1-Jag2—serotype 1-Jagged 2, SIRT1 KO—SIRT1 inducible knockout (details about knockout mice in [7]), Smad3—mothers against decapentaplegic homolog 3, Smad7—mothers against decapentaplegic homolog 7, SOD—superoxide dismutase, SU5416—Sugen, TGF-β1—transforming growth factor-β1, TnC—troponin C, TNF-α—tumor necrosis factor-α, tot.—totally, Trx-1—thioredoxin-1, TU—transduction units, VCAM-1—vascular cell adhesion molecule-1, w—week/weeks, α-SMA—alpha smooth muscle actin.

**Table 3:** Effects of sirtuin 1 (SIRT1) modulators on pulmonary artery endothelial and smooth muscle cells in vitro: impact on viability, regulation of apoptosis and proliferation, anti-inflammatory and antioxidant activity, mitochondrial dysfunction, and SIRT1 expression.

Cell Culture	Conditions	Modulator		Effects					Ref.
				Viability	Regulation of Apoptosis and Proliferation	Anti-Inflammatory, Antioxidant	Mitochondrial Dysfunction	SIRT1 Expression/ Other Mechanisms	
**HUMAN**									
iPAH PASMC obtained during lung transplantation	in normoxia	Stac-3; 10 μM		↔	↓ PCNA	↑ SOD2	↑ VDAC, PPARα, CS, ERRα, PGC-1α, GLUT1, LDH	↔ SIRT1 expression	[7]
iPAH PASMC obtained during lung transplantation	48 h in normoxia	SRT2104; 10 μM		n/d	↑ TSC2; ↓ Col1A1, p-Akt, fibronectin	n/d	n/d	-	[16]
PAEC	72 h in hypoxia (10% O_2_, 5% CO_2_)	SRT1720; 4 μM		↑	↑ p-Akt, Bcl2, HIF-1	↑ HIF-1	n/d	↔ Akt	[107]
		NAC; 5000 μM		n/d	↓ p-Akt, Bcl2, HIF-1	↓ HIF-1	n/d	↔ Akt	
PASMC	PDGF (10 ng/mL) for 48 h	resveratrol; 10 µM		n/d	↓ (proliferation)	↓ NF-κB	n/d	-	[156]
PASMC	48 h in hypoxia (1% O_2_)	scutellarein; 50 and 100 µM		↓↓	↓↓ (proliferation and apoptosis)	↓↓ IL-6; ↓ TNF-α, IL-1β	n/d	↑↑ SIRT1 expression	[157]
PASMC	PDGF (10 ng/mL) for 48 h	resveratrol	10 µM	n/d	↔ (proliferation and apoptosis)	n/d	n/d	-	[159]
			30 µM		↑ atrogin-1; ↔ (proliferation and apoptosis)				
			100 µM		↑ atrogin-1; ↓↓ (proliferation and apoptosis)				
PASMC	48 h in hypoxia	si-circSIRT1		n/d	↓ (proliferation) ↑ (apoptosis)	n/d	n/d	↓ migration, Beclin-1, ATG5, LC3 II, Akt3 ↑ p62, miR-145-5p	[168]
**RAT**									
PASMC	in normoxia	Stac-3; 10 μM		n/d	↓↓↓ ac-histone H1, ac-FOXO1; ↓ PCNA	n/d	↓ ac-PGC-1α	↔ SIRT1 expression	[7]
PASMC	24 h in hypoxia (3% O_2_)	circ-SIRT1		↓	↓ Smad3, Smad7, TGF-β1, α-SMA	↓ VCAM-1	n/d	↓ migration	[63]
PASMC	24 h in hypoxia (92% N_2_, 5% CO_2_, 3% O_2_)	resveratrol 30 and 50 μM		n/d	↓ (proliferation)	n/d	n/d	n/d	[108]
		SRT1720 (1, 3, 5, and 10 μM)		↓	↑↑ (apoptosis; 1 μM); ↓ (proliferation; 3, 5, and 10 μM)	n/d	n/d	↑ SNO; ↓ migration; ↓↓ mPT	
PASMC	24 h in hypoxia (5% CO_2_, 1% O_2_)	Rat 1-Jag2		↓	↑ Bax; ↓↓ Bcl2	n/d	↑↑ Tom20, Coxiv	n/d	[109]
PMEC	in hypoxia (5% CO_2_)	phoenixin 20	10 nM	n/d	n/d	↑ SOD; ↓ MDA, TNF-α, IL-6, NLRP3, ASC, MCP-1	n/d	↑ SIRT1 expression; ↓ MCP-1	[158]
			20 nM			↑↑ SOD; ↓↓ MDA, TNF-α, IL-6, NLRP3, ASC, MCP-1			
PASMC	48 h in hypoxia (2.5% O_2_, 5% CO_2_)	15-HETE		n/d	n/d	n/d	n/d	↑ SIRT1 expression	[160]
		15-HETE + serum deprivation		↑	↑ Bcl-xl, Bcl2; ↓ caspase 3 ↓ (apoptosis)	n/d	n/d	n/d	
PASMC	24 h in hypoxia (2% O_2_, 5% CO_2_)	Shionone	2 µg/ml	↔	↑ Bax; ↓↓ Bcl2; ↑↑ (apoptosis)	↓ TNF-α, IL-6; ↔ IL-1β	n/d	↑↑ SIRT1 expression ↑↑ eNOS; ↓ ET-1	[161]
			4 µg/ml	↓↓	↑↑ Bax; ↓↓ Bcl2; ↑↑ (apoptosis)	↓↓ TNF-α, IL-1β, IL-6	n/d	↑↑ SIRT1 expression ↑↑ eNOS; ↓↓ ET-1	
			8 µg/ml	↓↓	↑↑ Bax; ↓↓ Bcl2; ↑↑ (apoptosis)	↓↓ TNF-α, IL-1β, IL-6	n/d	↑↑ SIRT1 expression ↑↑ eNOS; ↓↓ ET-1	
PAEC	24 h in hypoxia (2% O_2_, 5% CO_2_)		2, 4, 8 µg/ml	n/d	n/d	n/d	n/d	↑↑ SIRT1 expression	
			8 µg/mL + SIRT1-siRNA	↓↓	↑↑ Bax; ↓↓ Bcl2; ↑↑ (apoptosis)	↑↑ TNF-α, IL-1β, IL-6	n/d	↓↓ eNOS; ↑↑ ET-1	

↑—increase, ↓—decrease, ↓/↑ *p* < 0.05, ↓↓/↑↑ *p* < 0.01, ↓↓↓ *p* < 0.001, ↔ no change, 1-Jag2—adeno-associated virus serotype 1-Jag2, ATG5—Autophagy-Related Gene 5, Akt—protein kinase B, ASC—apoptosis-associated speck-like protein containing a CARD, Bax—Bcl-2-associated x-protein, Bcl2—B-cell lymphoma 2, Col1A1—collagen 1 A1, Coxiv—cytochrome C oxidase subunit IV, CS—citrate synthase, ERRα—estrogen-related receptor alpha, FOXO1—Forkhead box protein O 1, GLUT1—glucose transporter 1, HIF-1—hypoxia-inducible factor-1, IL-1β—interleukin 1β, IL-6—interleukin-6, iPAH—idiopathic pulmonary arterial hypertension, LC3 II—Microtubule-Associated Protein 1 Light Chain 3 lipid-conjugated form, LDH—lactate dehydrogenase, MCP-1—monocyte chemoattractant protein-1, MDA—malondialdehyde, miR-145-5p—microRNA-145-5p, mPT—mitochondrial permeability transition, n/d—not determined, NAC—N-acetylcysteine, NF-κB—nuclear factor-kappa B, NLRP3—domains-containing protein 3, p62—SQSTM1/Sequestosome 1, PAEC—pulmonary artery endothelial cells, p-Akt—phosphorylated protein kinase B, PASMC—pulmonary artery smooth muscle cells, PCNA—proliferating cell nuclear antigen, PDGF—platelet-derived growth factor-BB, PGC-1α—peroxisome proliferator-activated receptor-gamma coactivator-1 alpha, PMEC—pulmonary microvascular endothelial cells, PPARα—peroxisome proliferator-activated receptor alpha, ref.—reference, si-circSIRT1—small interfering RNA of targeting circSIRT1, Smad3—mothers against decapentaplegic homolog 3, Smad7—mothers against decapentaplegic homolog 7, SNO—secondary necrosis, SOD—superoxide dismutase, TGF-β1—transforming growth factor-β1, TNF-α—tumor necrosis factor-α, Tom20—translocase of outer mitochondrial membrane 20, TSC2—tuberous sclerosis complex subunit 2, VCAM-1—vascular cell adhesion molecule-1, VDAC—voltage-dependent anion channel, α-SMA—α-smooth muscle actin.

## Data Availability

No new data were created or analyzed in this study. Data sharing is not applicable to this article.

## Abbreviations

The following abbreviations are used in this manuscript:

ac-eNOS	acetylated endothelial nitric synthase
Ach	acetylcholine
Ac-Lys	acetyl-lysine
ADP	adenosine diphosphate
Akt	protein kinase B
AMPK	adenosine monophosphate-activated kinase
ANP	atrial natriuretic peptide
APC	adenomatous polyposis coli
ASC	apoptosis-associated speck-like protein containing a CARD
ATP	adenosine triphosphate
Bax	Bcl-2-associated x-protein
Bcl-2	B-cell lymphoma-2
Bcl-xl	B-cell lymphoma-extra-large
BMP	bone morphogenetic protein
BNP	brain natriuretic peptide
cAMP	cyclic adenosine monophosphate
CCL5	CC Motif Chemokine Ligand 5
CD68	cluster of differentiation 68
CDK2	cyclin-dependent kinase 2
circ-SIRT1	circular RNA derived from the SIRT1 gene
CKIα	casein kinase I alpha
c-Myc	cellular myelocytomatosis oncogene
COL1A1	collagen type I alpha I chain
COX2	cyclooxygenase 2
CR	calorie restriction
CS	citrate synthase
CTGF	connective tissue growth factor
CVD	cardiovascular diseases
Drp1	dynamin-related protein 1
EC	endothelial cells
EC_1.5_	1.5-fold effective concentration
EC_50_	half-maximal effective concentration
EGFR	epidermal growth factor receptor
EMA	European Medicines Agency
EndMT	endothelial-to-mesenchymal transition
eNOS	endothelial nitric oxide synthase
ERRα	estrogen-related receptor alpha
ET	ejection time
ET-1	endothelin-1
FDA	Food and Drug Administration
FOXO1	Forkhead box protein O1
FOXO3a	Forkhead box protein O3a
G0	gap 0
G1	gap 1
Gclc	glutamate-cysteine ligase catalytic subunit
Gclm	glutamate-cysteine ligase modifier subunit
GLUT1	glucose transporter 1
gp91phox	glycoprotein 91-phagocyte oxidase
GSK3β	glycogen synthase kinase 3β
H1	histone H1
HFpEF	heart failure with preserved ejection fraction
HIFs	hypoxia-inducible factors
HIF-α	hypoxia-inducible factor-alpha
HMGB1	high-mobility group box 1
HO-1	heme oxygenase-1
HUVEC	human umbilical vein endothelial cells
I/R	ischemia/reperfusion
IC_50_	half-maximal inhibitory concentration
ICAM-1	intercellular adhesion molecule 1
IFN-γ	interferon-gamma
IIA-Fc	activin receptor type IIA fusion protein
IL-10	interleukin-10
IL-18	interleukin-18
IL-1β	interleukin-1beta
IL-2	interleukin-2
IL-4	interleukin-4
IL-6	interleukin-6
iNOS	inducible nitric oxide synthase
iPAH	idiopathic pulmonary arterial hypertension
IκBα	NF-κB inhibitor alpha
Keap1	Kelch-like ECH-associated protein 1
Kv1.5	voltage-gated potassium channel
LDH	lactate dehydrogenase
LEF	lymphoid enhancer-binding factor
L-NAME	N-nitro-L-arginine methyl ester
LRP	lipoprotein receptor-related protein
LV	left ventricle
MCP-1	monocyte chemotactic protein-1
MCT	monocrotaline
MDA	malondialdehyde
MI	myocardial infarction
miR	microRNA
MMP2	matrix metalloproteinase 2
MMP9	matrix metalloproteinase 9
MnSOD	manganese-dependent superoxide dismutase
mPAP	mean pulmonary arterial pressure
MPO	myeloperoxidase
mPT	mitochondrial permeability transition
MSC	mesenchymal stem cells
mTOR	mechanistic target of rapamycin
mTORC1	mechanistic target of rapamycin complex 1
mTORC2	mechanistic target of rapamycin complex 2
MuRF-1	muscle RING-finger protein-1
NAD^+^	nicotinamide adenine nucleotide
NADPH	nicotinamide adenine dinucleotide phosphate
NAM	nicotinamide
NAMPT	nicotinamide phosphoribosyltransferase
NF-κB	nuclear factor-kappa B
NLRP3	nucleotide-binding oligomerization domain-like receptor protein 3
NNT	nicotinamide nucleotide transhydrogenase
NO	nitric oxide
Notch1	neurogenic locus notch homolog protein 1
NOX 4	NADPH oxidase 4
NOX-1	NADPH oxidase-1
Nqo1	NAD(P)H: quinone oxidoreductase-1
Nrf2	nuclear factor erythroid 2-related factor 2
p21	cyclin-dependent kinase inhibitor 1A
p47phox	neutrophil cytosolic factor 1
p53	tumor protein
PA	pulmonary arteries
PAAT	pulmonary artery acceleration time
PAEC	pulmonary artery endothelial cells
PAH	pulmonary arterial hypertension
PAH-CTD	PAH associated with connective tissue disease
p-Akt	phosphorylated-protein kinase B
PASMC	pulmonary arterial smooth muscle cells
PCNA	proliferating cell nuclear antigen
PDGF	platelet-derived growth factor
p-eNOS	phosphorylated endothelial nitric oxide synthase
PGC-1α	peroxisome proliferator-activated receptor-gamma coactivator-1 alpha
PGI2	prostacyclin I2
PH	pulmonary hypertension
PI3K	phosphoinositide 3-kinase
PINK1	PTEN-induced putative kinase 1
p-JNK	phosphorylated Jun N-terminal kinase
PKA	cAMP-dependent protein kinase
PKIA	cAMP-dependent protein kinase inhibitor
PMEC	pulmonary microvascular endothelial cells
PPARα	peroxisome proliferator-activated receptor alpha
PTEN	phosphatase and tensin homologue deleted on chromosome 10
PTGS2	prostaglandin-endoperoxide synthase 2
ROS	reactive oxygen species
RV	right ventricle
RVH	right ventricle hypertrophy
RVSP	right ventricle systolic pressure
S	septum
SD	Sprague-Dawley
si-Jag2	short interfering RNA targeting Jagged2
Sir2	silent information regulator 2
SIRT1	sirtuin 1
SIRT2	sirtuin 2
SIRT3	sirtuin 3
SIRT7	sirtuin 7
SIRTs	sirtuins
SKL	secreted Klotho
Smad1/5/8	mothers against decapentaplegic homolog 1/5/8
Smad2/3	mothers against decapentaplegic homolog 2/3
Smad4	mothers against decapentaplegic homolog 4
Smad7	mothers against decapentaplegic homolog 7
SOD2	superoxide dismutase 2
SRC	non-receptor tyrosine kinase Src
STAT3	signal transducer and activator of transcription 3
SU5416	Sugen 5416
TAPSE	tricuspid annular plane systolic excursion
TCF	T-cell factor
TFAM	mitochondrial transcription factor
TGF-β	transforming growth factor-β
TnC	troponin C
TNF-α	tumor necrosis factor-alpha
Trx-1	thioredoxin-1
TSC2	tuberous sclerosis complex subunit 2
VCAM-1	vascular cell adhesion molecule 1
VDAC	voltage dependent anion channel
VEGFA	vascular endothelial growth factor A
VEGFR-2	vascular endothelial growth factor receptor 2
VSMC	vascular smooth muscle cells
Wnt	wingless-type mouse mammary tumor virus integration site family
YAP	yes-associated protein
α-SMA	alpha smooth muscle actin

## References

[1] Kovacs G., Bartolome S., Denton C.P., Gatzoulis M.A., Gu S., Khanna D., Badesch D., Montani D. (2024). Definition, classification and diagnosis of pulmonary hypertension. Eur. Respir. J..

[2] Guignabert C., Aman J., Bonnet S., Dorfmüller P., Olschewski A.J., Pullamsetti S., Rabinovitch M., Schermuly R.T., Humbert M., Stenmark K.R. (2024). Pathology and pathobiology of pulmonary hypertension: Current insights and future directions. Eur. Respir. J..

[3] Humbert M., McLaughlin V., Gibbs J.S.R., Gomberg-Maitland M., Hoeper M.M., Preston I.R., Souza R., Waxman A., Escribano Subias P., Feldman J. (2021). Sotatercept for the treatment of pulmonary arterial hypertension. N. Engl. J. Med..

[4] Hoeper M.M., Badesch D.B., Ghofrani H.A., Gibbs J.S.R., Gomberg-Maitland M., McLaughlin V.V., Preston I.R., Souza R., Waxman A.B., Grünig E. (2023). STELLAR Trial Investigators. Phase 3 trial of sotatercept for treatment of pulmonary arterial hypertension. N. Engl. J. Med..

[5] Campagna R., Vignini A. (2023). NAD^+^ homeostasis and NAD^+^-consuming enzymes: Implications for vascular health. Antioxidant.

[6] Zhou R., Barnes K., Gibson S., Fillmore N. (2024). Dual-edged role of SIRT1 in energy metabolism and cardiovascular disease. Am. J. Physiol. Heart Circ. Physiol..

[7] Zurlo G., Piquereau J., Moulin M., Pires Da Silva J., Gressette M., Ranchoux B., Garnier A., Ventura-Clapier R., Fadel E., Humbert M. (2018). Sirtuin 1 regulates pulmonary artery smooth muscle cell proliferation: Role in pulmonary arterial hypertension. J. Hypertens..

[8] Cheng X.W., Narisawa M., Jin X., Murohara T., Kuzuya M. (2018). Sirtuin 1 as a potential therapeutic target in pulmonary artery hypertension. J. Hypertens..

[9] Kurakula K., Smolders V., Tura-Ceide O., Jukema J.W., Quax P.H.A., Goumans M.J. (2021). Endothelial dysfunction in pulmonary hypertension: Cause or consequence?. Biomedicines.

[10] Zhu J., Yang L., Jia Y., Balistrieri A., Fraidenburg D.R., Wang J., Tang H., Yuan J.X. (2022). Pathogenic mechanisms of pulmonary arterial hypertension: Homeostasis imbalance of endothelium-derived relaxing and contracting factors. JACC Asia.

[11] Humbert M., Morrell N.W., Archer S.L., Stenmark K.R., MacLean M.R., Lang I.M., Christman B.W., Weir E.K., Eickelberg O., Voelkel N.F. (2004). Cellular and molecular pathobiology of pulmonary arterial hypertension. J. Am. Coll. Cardiol..

[12] Bousseau S., Sobrano Fais R., Gu S., Frump A., Lahm T. (2023). Pathophysiology and new advances in pulmonary hypertension. BMJ Med..

[13] Andersen S., Nielsen-Kudsk J.E., Vonk Noordegraaf A., de Man F.S. (2019). Right ventricular fibrosis. Circulation.

[14] Hu Y., Chi L., Kuebler W.M., Goldenberg N.M. (2020). Perivascular inflammation in pulmonary arterial hypertension. Cells.

[15] Xu D., Hu Y.H., Gou X., Li F.Y., Yang X.Y., Li Y.M., Chen F. (2022). Oxidative stress and antioxidative therapy in pulmonary arterial hypertension. Molecules.

[16] Shen Y., Goncharov D.A., Pena A., Baust J., Chavez Barragan A., Ray A., Rode A., Bachman T.N., Chang B., Jiang L. (2022). Cross-talk between TSC2 and the extracellular matrix controls pulmonary vascular proliferation and pulmonary hypertension. Sci. Signal..

[17] Liu S.F., Nambiar Veetil N., Li Q., Kucherenko M.M., Knosalla C., Kuebler W.M. (2022). Pulmonary hypertension: Linking inflammation and pulmonary arterial stiffening. Front. Immunol..

[18] Wang R.R., Yuan T.Y., Wang J.M., Chen Y.C., Zhao J.L., Li M.T., Fang L.H., Du G.H. (2022). Immunity and inflammation in pulmonary arterial hypertension: From pathophysiology mechanisms to treatment perspective. Pharmacol. Res..

[19] Li C., Liu P., Song R., Zhang Y., Lei S., Wu S. (2017). Immune cells and autoantibodies in pulmonary arterial hypertension. Acta Biochim. Biophys. Sin..

[20] Stacher E., Graham B.B., Hunt J.M., Gandjeva A., Groshong S.D., McLaughlin V.V., Jessup M., Grizzle W.E., Aldred M.A., Cool C.D. (2012). Modern age pathology of pulmonary arterial hypertension. Am. J. Respir. Crit. Care Med..

[21] Savai R., Pullamsetti S.S., Kolbe J., Bieniek E., Voswinckel R., Fink L., Scheed A., Ritter C., Dahal B.K., Vater A. (2012). Immune and inflammatory cell involvement in the pathology of idiopathic pulmonary arterial hypertension. Am. J. Respir. Crit. Care Med..

[22] Zhao H., Song J., Li X., Xia Z., Wang Q., Fu J., Miao Y., Wang D., Wang X. (2024). The role of immune cells and inflammation in pulmonary hypertension: Mechanisms and implications. Front. Immunol..

[23] Song J.L., Zheng S.Y., He R.L., Gui L.X., Lin M.J., Sham J.S.K. (2021). Serotonin and chronic hypoxic pulmonary hypertension activate a NADPH oxidase 4 and TRPM2 dependent pathway for pulmonary arterial smooth muscle cell proliferation and migration. Vascul. Pharmacol..

[24] Archer S.L. (2017). Pyruvate Kinase and Warburg Metabolism in Pulmonary Arterial Hypertension: Uncoupled Glycolysis and the Cancer-Like Phenotype of Pulmonary Arterial Hypertension. Circulation.

[25] Zhang W., Liu B., Wang Y., Zhang H., He L., Wang P., Dong M. (2022). Mitochondrial dysfunction in pulmonary arterial hypertension. Front. Physiol..

[26] Liu R., Xu C., Zhang W., Cao Y., Ye J., Li B., Jia S., Weng L., Liu Y., Liu L. (2022). FUNDC1-mediated mitophagy and HIF1α activation drives pulmonary hypertension during hypoxia. Cell Death Dis..

[27] Liang S., Yegambaram M., Wang T., Wang J., Black S.M., Tang H. (2022). Mitochondrial Metabolism, Redox, and Calcium Homeostasis in Pulmonary Arterial Hypertension. Biomedicines.

[28] Pokharel M.D., Marciano D.P., Fu P., Franco M.C., Unwalla H., Tieu K., Fineman J.R., Wang T., Black S.M. (2023). Metabolic reprogramming, oxidative stress, and pulmonary hypertension. Redox Biol..

[29] Ryanto G.R.T., Suraya R., Nagano T. (2023). Mitochondrial Dysfunction in Pulmonary Hypertension. Antioxidants.

[30] Ling H., Peng L., Wang J., Rahhal R., Seto E. (2018). Histone Deacetylase SIRT1 Targets Plk2 to Regulate Centriole Duplication. Cell Rep..

[31] Teixeira C.S.S., Cerqueira N.M.F.S.A., Gomes P., Sousa S.F. (2020). A Molecular Perspective on Sirtuin Activity. Int. J. Mol. Sci..

[32] Majeed Y., Halabi N., Madani A.Y., Engelke R., Bhagwat A.M., Abdesselem H., Agha M.V., Vakayil M., Courjaret R., Goswami N. (2021). SIRT1 promotes lipid metabolism and mitochondrial biogenesis in adipocytes and coordinates adipogenesis by targeting key enzymatic pathways. Sci. Rep..

[33] Wu Q.J., Zhang T.N., Chen H.H., Yu X.F., Lv J.L., Liu Y.Y., Liu Y.S., Zheng G., Zhao J.Q., Wei Y.F. (2022). The sirtuin family in health and disease. Signal Transduct. Target. Ther..

[34] Kassan M., Vikram A., Li Q., Kim Y.R., Kumar S., Gabani M., Liu J., Jacobs J.S., Irani K. (2017). MicroRNA-204 promotes vascular endoplasmic reticulum stress and endothelial dysfunction by targeting Sirtuin1. Sci. Rep..

[35] Lu C.L., Liao M.T., Hou Y.C., Fang Y.W., Zheng C.M., Liu W.C., Chao C.T., Lu K.C., Ng Y.Y. (2020). Sirtuin-1 and Its Relevance in Vascular Calcification. Int. J. Mol. Sci..

[36] Yu H., Gan D., Luo Z., Yang Q., An D., Zhang H., Hu Y., Ma Z., Zeng Q., Xu D. (2024). α-Ketoglutarate improves cardiac insufficiency through NAD+-SIRT1 signaling-mediated mitophagy and ferroptosis in pressure overload-induced mice. Mol. Med..

[37] Poljšak B., Kovač V., Špalj S., Milisav I. (2023). The Central Role of the NAD+ Molecule in the Development of Aging and the Prevention of Chronic Age-Related Diseases: Strategies for NAD+ Modulation. Int. J. Mol. Sci..

[38] Hwang E.S., Song S.B. (2017). Nicotinamide is an inhibitor of SIRT1 in vitro, but can be a stimulator in cells. Cell Mol. Life Sci..

[39] Zhang Q.J., Wang Z., Chen H.Z., Zhou S., Zheng W., Liu G., Wei Y.S., Cai H., Liu D.P., Liang C.C. (2008). Endothelium-specific overexpression of class III deacetylase SIRT1 decreases atherosclerosis in apolipoprotein E-deficient mice. Cardiovasc. Res..

[40] Zhou S., Chen H.Z., Wan Y.Z., Zhang Q.J., Wei Y.S., Huang S., Liu J.J., Lu Y.B., Zhang Z.Q., Yang R.F. (2011). Repression of P66Shc expression by SIRT1 contributes to the prevention of hyperglycemia-induced endothelial dysfunction. Circ. Res..

[41] Man A.W.C., Li H., Xia N. (2019). The Role of Sirtuin1 in Regulating Endothelial Function, Arterial Remodeling and Vascular Aging. Front. Physiol..

[42] Potente M., Ghaeni L., Baldessari D., Mostoslavsky R., Rossig L., Dequiedt F., Haendeler J., Mione M., Dejana E., Alt F.W. (2007). SIRT1 controls endothelial angiogenic functions during vascular growth. Genes Dev..

[43] Budbazar E., Rodriguez F., Sanchez J.M., Seta F. (2020). The Role of Sirtuin-1 in the Vasculature: Focus on Aortic Aneurysm. Front. Physiol..

[44] Mengozzi A., Costantino S., Paneni F., Duranti E., Nannipieri M., Mancini R., Lai M., La Rocca V., Puxeddu I., Antonioli L. (2022). Targeting SIRT1 Rescues Age- and Obesity-Induced Microvascular Dysfunction in Ex Vivo Human Vessels. Circ. Res..

[45] Bartoli-Leonard F., Wilkinson F.L., Schiro A., Serracino Inglott F., Alexander M.Y., Weston R. (2021). Loss of SIRT1 in diabetes accelerates DNA damage-induced vascular calcification. Cardiovasc. Res..

[46] Gorenne I., Kumar S., Gray K., Figg N., Yu H., Mercer J., Bennett M. (2013). Vascular smooth muscle cell sirtuin 1 protects against DNA damage and inhibits atherosclerosis. Circulation.

[47] Gao P., Xu T.T., Lu J., Li L., Xu J., Hao D.L., Chen H.Z., Liu D.P. (2014). Overexpression of SIRT1 in vascular smooth muscle cells attenuates angiotensin II-induced vascular remodeling and hypertension in mice. J Mol. Med..

[48] Chen H.Z., Wang F., Gao P., Pei J.F., Liu Y., Xu T.T., Tang X., Fu W.Y., Lu J., Yan Y.F. (2016). Age-Associated Sirtuin 1 Reduction in Vascular Smooth Muscle Links Vascular Senescence and Inflammation to Abdominal Aortic Aneurysm. Circ. Res..

[49] Kloza M., Krzyżewska A., Kozłowska H., Budziak S., Baranowska-Kuczko M. (2025). Empagliflozin Plays Vasoprotective Role in Spontaneously Hypertensive Rats via Activation of the SIRT1/AMPK Pathway. Cells.

[50] Wang A.J., Zhang J., Xiao M., Wang S., Wang B.J., Guo Y., Tang Y., Gu J. (2021). Molecular mechanisms of doxorubicin-induced cardiotoxicity: Novel roles of sirtuin 1-mediated signaling pathways. Cell Mol. Life Sci..

[51] Hsu C.P., Zhai P., Yamamoto T., Maejima Y., Matsushima S., Hariharan N., Shao D., Takagi H., Oka S., Sadoshima J. (2010). Silent information regulator 1 protects the heart from ischemia/reperfusion. Circulation.

[52] Kuno A., Hosoda R., Tsukamoto M., Sato T., Sakuragi H., Ajima N., Saga Y., Tada K., Taniguchi Y., Iwahara N. (2023). SIRT1 in the cardiomyocyte counteracts doxorubicin-induced cardiotoxicity via regulating histone H2AX. Cardiovasc. Res..

[53] Costantino S., Mengozzi A., Velagapudi S., Mohammed S.A., Gorica E., Akhmedov A., Mongelli A., Pugliese N.R., Masi S., Virdis A. (2023). Treatment with recombinant Sirt1 rewires the cardiac lipidome and rescues diabetes-related metabolic cardiomyopathy. Cardiovasc. Diabetol..

[54] Tang Y.J., Zhang Z., Yan T., Chen K., Xu G.F., Xiong S.Q., Wu D.Q., Chen J., Jose P.A., Zeng C.Y. (2024). Irisin attenuates type 1 diabetic cardiomyopathy by anti-ferroptosis via SIRT1-mediated deacetylation of p53. Cardiovasc. Diabetol..

[55] Qiu H., Sun Y., Wang X., Gong T., Su J., Shen J., Zhou J., Xia J., Wang H., Meng X. (2024). Lamin A/C deficiency-mediated ROS elevation contributes to pathogenic phenotypes of dilated cardiomyopathy in iPSC model. Nat. Commun..

[56] Yang Y., Liu Y., Wang Y., Chao Y., Zhang J., Jia Y., Tie J., Hu D. (2022). Regulation of SIRT1 and Its Roles in Inflammation. Front. Immunol..

[57] Wang F., Chen H.Z. (2020). Histone Deacetylase SIRT1, Smooth Muscle Cell Function, and Vascular Diseases. Front. Pharmacol..

[58] Ding Y.N., Wang H.Y., Chen X.F., Tang X., Chen H.Z. (2025). Roles of Sirtuins in Cardiovascular Diseases: Mechanisms and Therapeutics. Circ. Res..

[59] Singh C.K., Chhabra G., Ndiaye M.A., Garcia-Peterson L.M., Mack N.J., Ahmad N. (2018). The Role of Sirtuins in Antioxidant and Redox Signaling. Antioxid. Redox Signal..

[60] Liu Y.P., Wen R., Liu C.F., Zhang T.N., Yang N. (2023). Cellular and molecular biology of sirtuins in cardiovascular disease. Biomed. Pharmacother..

[61] Li Y., Kang K., Bao H., Liu S., Zhao B., Hu G., Wu J. (2025). Research Progress on the Interaction Between SIRT1 and Mitochondrial Biochemistry in the Aging of the Reproductive System. Biology.

[62] Mortuza R., Feng B., Chakrabarti S. (2015). SIRT1 reduction causes renal and retinal injury in diabetes through endothelin 1 and transforming growth factor β1. J. Cell Mol. Med..

[63] Diao W., Liu G., Shi C., Jiang Y., Li H., Meng J., Shi Y., Chang M., Liu X. (2022). Evaluating the Effect of Circ-Sirt1 on the Expression of SIRT1 and Its Role in Pathology of Pulmonary Hypertension. Cell Transplant..

[64] Zhan H., Huang F., Niu Q., Jiao M., Han X., Zhang K., Ma W., Mi S., Guo S., Zhao Z. (2021). Downregulation of miR-128 Ameliorates Ang II-Induced Cardiac Remodeling via SIRT1/PIK3R1 Multiple Targets. Oxidative Med. Cell. Longev..

[65] Wu B.W., Wu M.S., Liu Y., Lu M., Guo J.D., Meng Y.H., Zhou Y.H. (2021). SIRT1-mediated deacetylation of NF-κB inhibits the MLCK/MLC2 pathway and the expression of ET-1, thus alleviating the development of coronary artery spasm. Am. J. Physiol. Heart Circ. Physiol..

[66] Elmorsy E.A., Elashry H.A., Alkhamiss A.S., Alsaykhan H., Hamad R.S., Abdel-Reheim M.A., Alsoghair M., Alharbi M.S., Gabr A.M., Ellethy A.T. (2025). E1231/NMN protects against experimental metabolic syndrome: The central role of SIRT1 in modulating AKT/Nrf2/NFκB signaling. Front. Pharmacol..

[67] Breitenstein A., Stein S., Holy E.W., Camici G.G., Lohmann C., Akhmedov A., Spescha R., Elliott P.J., Westphal C.H., Matter C.M. (2011). Sirt1 inhibition promotes in vivo arterial thrombosis and tissue factor expression in stimulated cells. Cardiovasc. Res..

[68] López-Fernández-Sobrino R., Soliz-Rueda J.R., Ávila-Román J., Arola-Arnal A., Suárez M., Muguerza B., Bravo F.I. (2021). Blood Pressure-Lowering Effect of Wine Lees Phenolic Compounds Is Mediated by Endothelial-Derived Factors: Role of Sirtuin 1. Antioxidants.

[69] Kim K.T., Heo J.B., Roh T., Jeon S.M., Heo H.J., Choi Y.J., Jo E.K., Song G.Y., Paik S. (2025). Resveratrol derivative SH-707 inhibits NLRP3 inflammasome activation via a sirtuin 1-dependent pathway. Int. Immunopharmacol..

[70] Chen X., Yang Q., Shen Y., Hou J., Yuan Q., Zhong Z., Liu Y. (2023). Research progress on the role of exosomes in the pathogenesis, diagnosis, and treatment of pulmonary hypertension. Respir. Res..

[71] Yang H., Zhang W., Guo S., Zhang M., Hu L., Li X., Liu J., Wang J., Yin Y. (2023). Progress of pyroptosis in pulmonary hypertension. Heart Fail. Rev..

[72] Wang H., Zhao Q., Zhang Y., Li T., Zhao M., Yang W., Wang G. (2022). The role of ferroptosis in pulmonary hypertension. Front. Pharmacol..

[73] Zhao L., Li M., Hu H., Liu Z., Zhang Y. (2022). Mitochondria and mitochondrial regulators in the development of pulmonary hypertension. Front. Med..

[74] Li Y., Yang Y., Luo J., Włodarski P.K., Wang G. (2022). Molecular mechanisms involved in the development of pulmonary arterial hypertension (PAH). J. Physiol. Pharmacol..

[75] Włodarski A., Strycharz J., Wróblewski A., Kasznicki J., Drzewoski J., Śliwińska A. (2020). The Role of microRNAs in Metabolic Syndrome-Related Oxidative Stress. Int. J. Mol. Sci..

[76] Jin Q., Xie X., Wang C., Zhang W., Wang T., Li S., Yang Y., Li J., Zhang H. (2021). Endothelial cell metabolism in pulmonary arterial hypertension. Front. Pharmacol..

[77] Zhang Y., He J., Wang Y., Chen H., Lin J., Zhong C., Li L., Huang J., Wang H., Liang G. (2023). Mitochondrial metabolic reprogramming-mediated immunogenic cell death reveals immune and prognostic features of pulmonary arterial hypertension. Front. Immunol..

[78] Ho M.F., Walseth T.F., Anderson S.M., Gerrity R., Hohmeier K.C., Johnson L.W., Croatt A.J., Nath K.A., Limper A.H., Leof E.B. (2021). Increased CD38 in the lungs of patients with pulmonary arterial hypertension. Am. J. Physiol. Lung Cell Mol. Physiol..

[79] Waldman M., Nudelman V., Shainberg A., Kornwoski R., Aravot D., Abraham N.G., Arad M., Hochhauser E. (2008). Regulation of oxidative stress and apoptosis in pulmonary hypertension: The role of heme oxygenase-1. J. Cardiovasc. Pharmacol..

[80] Hou L., Guo D., Wang D., He W., Wu C., Yang J. (2023). Research progress on the mechanism of vascular remodeling in pulmonary hypertension: The role of endothelial cells and endothelial microenvironment. Biomed. Pharmacother..

[81] Guo J., Wang Y., Guo H., Zhou S., Wu B., Zhu L., Wang Y. (2022). Molecular mechanisms of vascular remodeling in idiopathic pulmonary arterial hypertension. Front. Pharmacol..

[82] Fan C., Luo Y., Ma Y., Chen Y., Li X., Yang W., Yang X., Li W., Sun L. (2022). Epigenetics in pulmonary hypertension: Mechanisms and therapeutic targets. Front. Genet..

[83] Scisciola L., Fusi F., Iside C., Fiore D., Liccardo D., Iannone M., Marfella R., Barbieri M., Paolisso G., D’Onofrio N. (2022). Epigenetic mechanisms in pulmonary arterial hypertension: The key to precision medicine. Front. Cardiovasc. Med..

[84] Fusi J., Bianchi S., Daniele S., Pellegrini S., Martini C., Galetta F., Giovannini L., Franzoni F. (2018). An in vitro comparative study of the antioxidant activity and SIRT1 modulation of natural compounds. Biomed. Pharmacother..

[85] Iside C., Scafuro M., Nebbioso A., Altucci L. (2020). SIRT1 Activation by Natural Phytochemicals: An Overview. Front. Pharmacol..

[86] Kimira Y., Kasahara Y., Matsubara H. (2023). Current understanding and future therapeutic prospects for pulmonary arterial hypertension with BMPR2 mutations. Int. J. Mol. Sci..

[87] Baskaran D., Suresh K. (2023). Pulmonary hypertension: Biomarkers and role of microRNAs. Am. J. Physiol. Lung Cell Mol. Physiol..

[88] Ho J.H., Baskaran R., Wang M.F., Mohammedsaleh Z.M., Yang H.S., Balasubramanian B., Lin W.T. (2022). Dipeptide IF and exercise training attenuate hypertension in SHR rats by inhibiting fibrosis and hypertrophy and activating AMPKα1, SIRT1, and PGC1α. Int. J. Mol. Sci..

[89] Galiniak S., Walczak M., Wilińska M., Kukla M., Michalski Ł., Biesiada G. (2021). Role of microRNAs in pulmonary arterial hypertension: Review and exploratory analysis. Biomedicines.

[90] Kumar A., Sharma R., Rehman M.U., Shah B.A., Goyal S.N. (2022). Pharmacological overview of microRNA-based drugs for pulmonary arterial hypertension. Naunyn-Schmiedebergs Arch. Pharmacol..

[91] Sharifi-Rad J., Rayess Y.E., Rizk A.A., Sadaka C., Zgheib R., Zam W., Sestito S., Rapposelli S., Neffe-Skocińska K., Zielińska D. (2020). Turmeric and its major compound curcumin on health: Bioactive effects and safety profiles for food, pharmaceutical, biotechnological and medicinal applications. Front. Pharmacol..

[92] Neag M.A., Mocan A., Echeverría J., Pop R.M., Bocsan C.I., Crişan G., Buzoianu A.D. (2018). Berberine: Botanical occurrence, traditional uses, extraction methods, and relevance in cardiovascular, metabolic, hepatic, and renal disorders. Front. Pharmacol..

[93] Park S.J., Ahmad F., Philp A., Baar K., Williams T., Luo H., Ke H., Rehmann H., Taussig R., Brown A.L. (2012). Resveratrol ameliorates aging-related metabolic phenotypes by inhibiting cAMP phosphodiesterases. Cell.

[94] Houghton M.J., Kerimi A., Tumova S., Boyle J.P., Williamson G. (2018). Quercetin preserves redox status and stimulates mitochondrial function in metabolically-stressed HepG2 cells. Free Radic. Biol. Med..

[95] Zheng Y., Kou J., Wang P., Ye T., Wang Z., Gao Z., Cong L., Li M., Dong B., Yang W. (2021). Berberine-induced TFEB deacetylation by SIRT1 promotes autophagy in peritoneal macrophages. Aging.

[96] Guo F., Xu F., Li S., Zhang Y., Lv D., Zheng L., Gan Y., Zhou M., Zhao K., Xu S. (2024). Amifostine ameliorates bleomycin-induced murine pulmonary fibrosis via NAD^+^/SIRT1/AMPK pathway-mediated effects on mitochondrial function and cellular metabolism. Eur. J. Med. Res..

[97] Jadeja R.N., Powell F.L., Jones M.A., Fuller J., Joseph E., Thounaojam M.C., Bartoli M., Martin P.M. (2018). Loss of NAMPT in aging retinal pigment epithelium reduces NAD^+^ availability and promotes cellular senescence. Aging.

[98] Cantó C., Gerhart-Hines Z., Feige J.N., Lagouge M., Noriega L., Milne J.C., Elliott P.J., Puigserver P., Auwerx J. (2009). AMPK regulates energy expenditure by modulating NAD^+^ metabolism and SIRT1 activity. Nature.

[99] Carollo C., Sorce A., Cirafici E., Mulè G., Caimi G. (2025). Sirtuins and resveratrol in cardiorenal diseases: A narrative review of mechanisms and therapeutic potential. Nutrients.

[100] Pacholec M., Bleasdale J.E., Chrunyk B., Cunningham D., Flynn D., Garofalo R.S., Griffith D., Griffor M., Loulakis P., Pabst B. (2010). SRT1720, SRT2183, SRT1460, and resveratrol are not direct activators of SIRT1. J. Biol. Chem..

[101] Villalba J.M., Alcaín F.J. (2012). Sirtuin activators and inhibitors. Biofactors.

[102] Milne J.C., Lambert P.D., Schenk S., Carney D.P., Smith J.J., Gagne D.J., Jin L., Boss O., Perni R.B., Vu C.B. (2007). Small molecule activators of SIRT1 as therapeutics for the treatment of type 2 diabetes. Nature.

[103] Zhu C., Dong X., Wang X., Zheng Y., Qiu J., Peng Y., Xu J., Chai Z., Liu C. (2022). Multiple roles of SIRT2 in regulating physiological and pathological signal transduction. Genet. Res. (Camb.).

[104] Murugasamy K., Munjal A., Sundaresan N.R. (2022). Emerging roles of SIRT3 in cardiac metabolism. Front. Cardiovasc. Med..

[105] Ma H., Yu Y., Mo L., Chen Q., Dong H., Xu Y., Zhuan B. (2023). Exosomal miR-663b from “M1” macrophages promotes pulmonary artery vascular smooth muscle cell dysfunction through inhibiting the AMPK/Sirt1 axis. Aging.

[106] Varshney R., Ali Q., Wu C., Sun Z. (2016). Monocrotaline-Induced Pulmonary Hypertension Involves Downregulation of Antiaging Protein Klotho and eNOS Activity. Hypertension.

[107] Xi L., Ruan L., Yao X., Zhang D., Yuan H., Li Q., Yan C. (2020). SIRT1 promotes pulmonary artery endothelial cell proliferation by targeting the Akt signaling pathway. Exp. Ther. Med.

[108] Yu L., Tu Y., Jia X., Fang K., Liu L., Wan L., Xiang C., Wang Y., Sun X., Liu T. (2017). Resveratrol Protects Against Pulmonary Arterial Hypertension in Rats via Activation of Silent Information Regulator 1. Cell Physiol. Biochem..

[109] Liu H., Pan Z., Wu X., Gong C., Hu J. (2024). Jagged 2 inhibition attenuates hypoxia-induced mitochondrial damage and pulmonary hypertension through Sirtuin 1 signaling. PLoS ONE.

[110] Ding M., Lei J., Qu Y., Zhang H., Xin W., Ma F., Liu S., Li Z., Jin F., Fu E. (2015). Calorie Restriction Attenuates Monocrotaline-induced Pulmonary Arterial Hypertension in Rats. J. Cardiovasc. Pharmacol..

[111] Zhou S., Li M.T., Jia Y.Y., Liu J.J., Wang Q., Tian Z., Liu Y.T., Chen H.Z., Liu D.P., Zeng X.F. (2015). Regulation of Cell Cycle Regulators by SIRT1 Contributes to Resveratrol-Mediated Prevention of Pulmonary Arterial Hypertension. Biomed. Res. Int..

[112] Vázquez-Garza E., Bernal-Ramírez L., Jerjes-Sánchez C., Lozano O., Acuña-Morín E., Vanoye-Tamez M., Ramos-González M.R., Chapoy-Villanueva H., Pérez-Plata L., Sánchez-Trujillo L. (2020). Resveratrol Prevents Right Ventricle Remodeling and Dysfunction in Monocrotaline-Induced Pulmonary Arterial Hypertension with a Limited Improvement in the Lung Vasculature. Oxidative Med. Cell. Longev..

[113] Hoffmann E., Wald J., Lavu S., Roberts J., Beaumont C., Haddad J., Elliott P., Westphal C., Jacobson E. (2013). Pharmacokinetics and tolerability of SRT2104, a first-in-class small molecule activator of SIRT1, after single and repeated oral administration in man. Br. J. Clin. Pharmacol..

[114] Miranda M.X., van Tits L.J., Lohmann C., Arsiwala T., Winnik S., Tailleux A., Stein S., Gomes A.P., Suri V., Ellis J.L. (2015). The Sirt1 activator SRT3025 provides atheroprotection in Apoe-/- mice by reducing hepatic Pcsk9 secretion and enhancing Ldlr expression. Eur. Heart J..

[115] Solomon J.M., Pasupuleti R., Xu L., McDonagh T., Curtis R., DiStefano P.S., Huber L.J. (2006). Inhibition of SIRT1 catalytic activity increases p53 acetylation but does not alter cell survival following DNA damage. Mol. Cell. Biol..

[116] Rye P.T., Frick L.E., Ozbal C.C., Lamarr W.A. (2011). Advances in label-free screening approaches for studying sirtuin-mediated deacetylation. J. Biomol. Screen..

[117] Kahyo T., Ichikawa S., Hatanaka T., Yamada M.K., Setou M. (2008). A novel chalcone polyphenol inhibits the deacetylase activity of SIRT1 and cell growth in HEK293T cells. J. Pharmacol. Sci..

[118] Grozinger C.M., Chao E.D., Blackwell H.E., Moazed D., Schreiber S.L. (2001). Identification of a class of small molecule inhibitors of the sirtuin family of NAD-dependent deacetylases by phenotypic screening. J. Biol. Chem..

[119] Lain S., Hollick J.J., Campbell J., Staples O.D., Higgins M., Aoubala M., McCarthy A., Appleyard V., Murray K.E., Baker L. (2008). Discovery, in vivo activity, and mechanism of action of a small-molecule p53 activator. Cancer Cell.

[120] Trapp J., Meier R., Hongwiset D., Kassack M.U., Sippl W., Jung M. (2007). Structure-activity studies on suramin analogues as inhibitors of NAD+-dependent histone deacetylases (sirtuins). ChemMedChem.

[121] Bononi G., Citi V., Martelli A., Poli G., Tuccinardi T., Granchi C., Testai L., Calderone V., Minutolo F. (2023). Sirtuin 1-activating derivatives belonging to the anilinopyridine class displaying in vivo cardioprotective activities. RSC Med. Chem..

[122] Campagna R., Mazzanti L., Pompei V., Alia S., Vignini A., Emanuelli M. (2024). The Multifaceted Role of Endothelial Sirt1 in Vascular Aging: An Update. Cells.

[123] Guo Y., Xu C., Man A.W.C., Bai B., Luo C., Huang Y., Xu A., Vanhoutte P.M., Wang Y. (2019). Endothelial SIRT1 prevents age-induced impairment of vasodilator responses by enhancing the expression and activity of soluble guanylyl cyclase in smooth muscle cells. Cardiovasc. Res..

[124] Lu C., Zhao H., Liu Y., Yang Z., Yao H., Liu T., Gou T., Wang L., Zhang J., Tian Y. (2023). Novel Role of the SIRT1 in Endocrine and Metabolic Diseases. Int. J. Biol. Sci..

[125] Toulassi I.A., Al Saedi U.A., Gutlapalli S.D., Poudel S., Kondapaneni V., Zeb M., Cancarevic I. (2021). A Paradigm Shift in the Management of Atherosclerosis: Protective Role of Sirtuins in Atherosclerosis. Cureus.

[126] Zhang T., Xu L., Guo X., Tao H., Liu Y., Liu X., Zhang Y., Meng X. (2024). The potential of herbal drugs to treat heart failure: The roles of Sirt1/AMPK. J. Pharm. Anal..

[127] Chen L., Li S., Zhu J., You A., Huang X., Yi X., Xue M. (2021). Mangiferin prevents myocardial infarction-induced apoptosis and heart failure in mice by activating the Sirt1/FoxO3a pathway. J. Cell. Mol. Med..

[128] Ding X., Zhu C., Wang W., Li M., Ma C., Gao B. (2024). SIRT1 is a regulator of autophagy: Implications for the progression and treatment of myocardial ischemia-reperfusion. Pharmacol. Res..

[129] Podyacheva E., Toropova Y. (2022). SIRT1 activation and its effect on intercalated disc proteins as a way to reduce doxorubicin cardiotoxicity. Front Pharmacol.

[130] Hu C., Zhang X., Teng T., Ma Z.G., Tang Q.Z. (2022). Cellular Senescence in Cardiovascular Diseases: A Systematic Review. Aging Dis..

[131] Zheng S., Yang L., Dai Q., Li X., Masuoka T., Lv J. (2025). Role of sirtuin 1 in depression-induced coronary heart disease: Molecular pathways and therapeutic potential (Review). Biomed. Rep..

[132] Fang W.J., Wang C.J., He Y., Zhou Y.L., Peng X.D., Liu S.K. (2018). Resveratrol alleviates diabetic cardiomyopathy in rats by improving mitochondrial function through PGC-1α deacetylation. Acta Pharmacol. Sin..

[133] Cheng H.L., Mostoslavsky R., Saito S., Manis J.P., Gu Y., Patel P., Bronson R., Appella E., Alt F.W., Chua K.F. (2003). Developmental defects and p53 hyperacetylation in Sir2 homolog (SIRT1)-deficient mice. Proc. Natl. Acad. Sci. USA.

[134] Wang W., Li Y., Zhang Y., Ye T., Wang K., Li S., Zhang Y. (2023). SIRT1 mediates the inhibitory effect of Dapagliflozin on EndMT by inhibiting the acetylation of endothelium Notch1. Cardiovasc. Diabetol..

[135] Mattagajasingh I., Kim C.S., Naqvi A., Yamamori T., Hoffman T.A., Jung S.B., DeRicco J., Kasuno K., Irani K. (2007). SIRT1 promotes endothelium-dependent vascular relaxation by activating endothelial nitric oxide synthase. Proc. Natl. Acad. Sci. USA.

[136] Pan Q., Gao Z., Zhu C., Peng Z., Song M., Li L. (2020). Overexpression of histone deacetylase SIRT1 exerts an antiangiogenic role in diabetic retinopathy via miR-20a elevation and YAP/HIF1α/VEGFA depletion. Am. J. Physiol. Endocrinol. Metab..

[137] Chen C., Hu S., Hu H.J., Liu Z.X., Wu X.T., Zou T., Su H. (2024). Dronedarone Attenuates Ang II-Induced Myocardial Hypertrophy Through Regulating SIRT1/FOXO3/PKIA Axis. Korean Circ. J..

[138] Yang J., Li L., Zheng X., Lu Z., Zhou H. (2023). Dapagliflozin attenuates myocardial hypertrophy via activating the SIRT1/HIF-1α signaling pathway. Biomed. Pharmacother..

[139] Jiang Q., Lu M., Li J., Zhu Z. (2021). Ginkgolide B Protects Cardiomyocytes from Angiotensin II-Induced Hypertrophy via Regulation of Autophagy through SIRT1-FoxO1. Cardiovasc. Ther..

[140] Zhang Y., Connelly K.A., Thai K., Wu X., Kapus A., Kepecs D., Gilbert R.E. (2017). Sirtuin 1 Activation Reduces Transforming Growth Factor-β1-Induced Fibrogenesis and Affords Organ Protection in a Model of Progressive, Experimental Kidney and Associated Cardiac Disease. Am. J. Pathol..

[141] Yin B., Wang Y.B., Li X., Hou X.W. (2022). β-aminoisobutyric acid ameliorates hypertensive vascular remodeling via activating the AMPK/SIRT1 pathway in VSMCs. Bioengineered.

[142] Bugyei-Twum A., Ford C., Civitarese R., Seegobin J., Advani S.L., Desjardins J.F., Kabir G., Zhang Y., Mitchell M., Switzer J. (2018). Sirtuin 1 activation attenuates cardiac fibrosis in a rodent pressure overload model by modifying Smad2/3 transactivation. Cardiovasc. Res..

[143] Gao D., Zuo Z., Tian J., Ali Q., Lin Y., Lei H., Sun Z. (2016). Activation of SIRT1 Attenuates Klotho Deficiency-Induced Arterial Stiffness and Hypertension by Enhancing AMP-Activated Protein Kinase Activity. Hypertension.

[144] Wang X., Yan J., Ni X., Hu S., Zhang M., Ying Y. (2023). Phloretin targets SIRT1 to alleviate oxidative stress, apoptosis, and inflammation in deep venous thrombosis. Toxicol. Res..

[145] Pai P.Y., Wong J.K.S., Cui Z.Y., Lin Y.Y., Lee S.D. (2022). Angiotensin II Receptor Blocker Irbesartan Enhanced SIRT1 longevity Signaling Replaces the Mitochondrial Biogenetic Survival Pathway to Attenuate Hypertension-Induced Heart Apoptosis. J. Cardiovasc. Dev. Dis..

[146] Han Y., Sun W., Ren D., Zhang J., He Z., Fedorova J., Sun X., Han F., Li J. (2020). SIRT1 agonism modulates cardiac NLRP3 inflammasome through pyruvate dehydrogenase during ischemia and reperfusion. Redox Biol..

[147] Ni Y., Deng J., Liu X., Li Q., Zhang J., Bai H., Zhang J. (2021). Echinacoside reverses myocardial remodeling and improves heart function via regulating SIRT1/FOXO3a/MnSOD axis in HF rats induced by isoproterenol. J. Cell. Mol. Med..

[148] Zhu H.Z., Zhang L.Y., Zhai M.E., Xia L., Cao Y., Xu L., Li K.F., Jiang L.Q., Shi H., Li X. (2021). GDF11 Alleviates Pathological Myocardial Remodeling in Diabetic Cardiomyopathy Through SIRT1-Dependent Regulation of Oxidative Stress and Apoptosis. Front. Cell Dev. Biol..

[149] Hao Z., Xu G., Yuan M., Tan R., Xia Y., Liu Y., Yin X. (2022). Leucine Supplementation in Middle-Aged Male Mice Improved Aging-Induced Vascular Remodeling and Dysfunction via Activating the Sirt1-Foxo1 Axis. Nutrients.

[150] Yang K., Velagapudi S., Akhmedov A., Kraler S., Lapikova-Bryhinska T., Schmiady M.O., Wu X., Geng L., Camici G.G., Xu A. (2023). Chronic SIRT1 supplementation in diabetic mice improves endothelial function by suppressing oxidative stress. Cardiovasc Res..

[151] Corbi G., Conti V., Troisi J., Colucci A., Manzo V., Di Pietro P., Calabrese M.C., Carrizzo A., Vecchione C., Ferrara N. (2019). Cardiac rehabilitation increases SIRT1 activity and β-hydroxybutyrate levels and decreases oxidative stress in patients with HF with preserved ejection fraction. Oxidative Med. Cell. Longev..

[152] Pei J., Liu Z., Wang C., Chu N., Liu L., Tang Y., Liu H., Xiang Q., Cheng H., Li M. (2022). Progesterone Attenuates SIRT1-Deficiency-Mediated Pre-Eclampsia. Biomolecules.

[153] Sasaki Y., Ikeda Y., Miyauchi T., Uchikado Y., Akasaki Y., Ohishi M. (2020). Estrogen-SIRT1 Axis Plays a Pivotal Role in Protecting Arteries Against Menopause-Induced Senescence and Atherosclerosis. J. Atheroscler. Thromb..

[154] Karolczak K., Watala C. (2023). Estradiol as the Trigger of Sirtuin-1-Dependent Cell Signaling with a Potential Utility in Anti-Aging Therapies. Int. J. Mol. Sci..

[155] Bendale D.S., Karpe P.A., Chhabra R., Shete S.P., Shah H., Tikoo K. (2013). 17-β Oestradiol prevents cardiovascular dysfunction in post-menopausal metabolic syndrome by affecting SIRT1/AMPK/H3 acetylation. Br. J. Pharmacol..

[156] Csiszar A., Labinskyy N., Olson S., Pinto J.T., Gupte S., Wu J.M., Hu F., Ballabh P., Podlutsky A., Losonczy G. (2009). Resveratrol prevents monocrotaline-induced pulmonary hypertension in rats. Hypertension.

[157] Tang H., Ning K., Wu B., Wang X., He J., Li P., Pan L., Zhang J., He Y., Bian S. (2025). Scutellarein ameliorates pulmonary arterial hypertension via sirtuin 1 mediated deacetylation of nicotinamide nucleotide transhydrogenase. Biochem. Pharmacol..

[158] Chai Y., Gu X., Zhang H., Xu X., Chen L. (2024). Phoenixin 20 ameliorates pulmonary arterial hypertension via inhibiting inflammation and oxidative stress. Aging.

[159] Paffett M.L., Lucas S.N., Campen M.J. (2012). Resveratrol reverses monocrotaline-induced pulmonary vascular and cardiac dysfunction: A potential role for atrogin-1 in smooth muscle. Vascul Pharmacol..

[160] Li F., You Y., Zhu H. (2018). 15-HETE protects pulmonary artery smooth muscle cells against apoptosis via SIRT1 regulation during hypoxia. Biomed. Pharmacother..

[161] Jiang Y., Hei B., Hao W., Lin S., Liu X., Meng X., Wang Y., Zhao M., Yu H., Yang L. (2024). Shionone Exhibits Anti-inflammatory and Antiproliferative Effects in Pulmonary Arterial Endothelial Cells and Smooth Muscle Cells via SIRT1 in Pulmonary Arterial Hypertension. Rev. Bras. Farmacogn..

[162] Boucherat O., Agrawal V., Lawrie A., Bonnet S. (2022). The Latest in Animal Models of Pulmonary Hypertension and Right Ventricular Failure. Circ. Res..

[163] Bueno-Beti C., Sassi Y., Hajjar R.J., Hadri L. (2018). Pulmonary Artery Hypertension Model in Rats by Monocrotaline Administration. Methods Mol. Biol..

[164] Corssac G.B., Bonetto J.P., Campos-Carraro C., Cechinel L.R., Zimmer A., Parmeggiani B., Grings M., Carregal V.M., Massensini A.R., Siqueira I. (2021). Pulmonary arterial hypertension induces the release of circulating extracellular vesicles with oxidative content and alters redox and mitochondrial homeostasis in the brains of rats. Hypertens. Res..

[165] Wu X.H., Ma J.L., Ding D., Ma Y.J., Wei Y.P., Jing Z.C. (2022). Experimental animal models of pulmonary hypertension: Development and challenges. Anim. Models Exp. Med..

[166] Corboz M.R., Nguyen T.L., Stautberg A., Cipolla D., Perkins W.R., Chapman R.W. (2024). Current Overview of the Biology and Pharmacology in Sugen/Hypoxia-Induced Pulmonary Hypertension in Rats. J. Aerosol Med. Pulm. Drug Deliv..

[167] Lin Q., Fan C., Skinner J.T., Hunter E.N., Macdonald A.A., Illei P.B., Yamaji-Kegan K., Johns R.A. (2019). RELMα Licenses Macrophages for Damage-Associated Molecular Pattern Activation to Instigate Pulmonary Vascular Remodeling. J. Immunol..

[168] Jing X., Wu S., Liu Y., Wang H., Huang Q. (2022). Circular RNA Sirtuin1 represses pulmonary artery smooth muscle cell proliferation, migration and autophagy to ameliorate pulmonary hypertension via targeting microRNA-145-5p/protein kinase-B3 axis. Bioengineered.

[169] Wilson D.N., Schacht S.E., Al-Nakkash L., Babu J.R., Broderick T.L. (2016). Resveratrol prevents pulmonary trunk remodeling but not right ventricular hypertrophy in monocrotaline-induced pulmonary hypertension. Pathophysiology.

[170] Klinke A., Berghausen E., Friedrichs K., Molz S., Lau D., Remane L., Berlin M., Kaltwasser C., Adam M., Mehrkens D. (2018). Myeloperoxidase aggravates pulmonary arterial hypertension by activation of vascular Rho-kinase. JCI Insight.

[171] Yeung F., Hoberg J.E., Ramsey C.S., Keller M.D., Jones D.R., Frye R.A., Mayo M.W. (2004). Modulation of NF-kappaB-dependent transcription and cell survival by the SIRT1 deacetylase. EMBO J..

[172] Yu Q., Dong L., Li Y., Liu G. (2018). SIRT1 and HIF1α signaling in metabolism and immune responses. Cancer Lett..

[173] Planavila A., Iglesias R., Giralt M., Villarroya F. (2011). Sirt1 acts in association with PPARα to protect the heart from hypertrophy, metabolic dysregulation, and inflammation. Cardiovasc. Res..

[174] Zhang Y., Li Y., Li J., Li B., Chong Y., Zheng G., Sun S., Feng F. (2019). SIRT1 alleviates isoniazid-induced hepatocyte injury by reducing histone acetylation in the IL-6 promoter region. Int. Immunopharmacol..

[175] Bai M., Lu C., An L., Gao Q., Xie W., Miao F., Chen X., Pan Y., Wang Q. (2020). SIRT1 relieves Necrotizing Enterocolitis through inactivation of Hypoxia-inducible factor (HIF)-1a. Cell Cycle.

[176] Alcendor R.R., Gao S., Zhai P., Zablocki D., Holle E., Yu X., Tian B., Wagner T., Vatner S.F., Sadoshima J. (2007). Sirt1 regulates aging and resistance to oxidative stress in the heart. Circ. Res..

[177] Elibol B., Kilic U. (2018). High Levels of SIRT1 Expression as a Protective Mechanism Against Disease-Related Conditions. Front. Endocrinol..

[178] Salla M., Karaki N., El Kaderi B., Ayoub A.J., Younes S., Abou Chahla M.N., Baksh S., El Khatib S. (2024). Enhancing the Bioavailability of Resveratrol: Combine It, Derivatize It, or Encapsulate It?. Pharmaceutics.

[179] Chimento A., De Amicis F., Sirianni R., Sinicropi M.S., Puoci F., Casaburi I., Saturnino C., Pezzi V. (2019). Progress to Improve Oral Bioavailability and Beneficial Effects of Resveratrol. Int. J. Mol. Sci..

[180] Wang J., Liu T., Chen P., Yin D., Zhang H., Qiu X., Zou S., Li W. (2025). Pharmacokinetic evaluation of two oral Resveratrol formulations in a randomized, open-label, crossover study in healthy fasting subjects. Sci. Rep..

[181] Patel K.R., Scott E., Brown V.A., Gescher A.J., Steward W.P., Brown K. (2011). Clinical trials of resveratrol. Ann. N. Y. Acad. Sci..

[182] Almeida L., Vaz-da-Silva M., Falcão A., Soares E., Costa R., Loureiro A.I., Fernandes-Lopes C., Rocha J.F., Nunes T., Wright L. (2009). Pharmacokinetic and safety profile of trans-resveratrol in a rising multiple-dose study in healthy volunteers. Mol. Nutr. Food Res..

[183] Tsai H.Y., Ho C.T., Chen Y.K. (2017). Biological actions and molecular effects of resveratrol, pterostilbene, and 3′-hydroxypterostilbene. J. Food Drug Anal..

[184] Reddy R., Kalra S.S., Alzghoul B., Khan A., Zayed Y. (2023). Effect of Obesity on Mortality in Pulmonary Hypertension-A Systematic Review and Meta-Analysis. J. Cardiovasc. Dev. Dis..

[185] Curjuric I., Imboden M., Bridevaux P.O., Gerbase M.W., Haun M., Keidel D., Kumar A., Pons M., Rochat T., Schikowski T. (2016). Common SIRT1 variants modify the effect of abdominal adipose tissue on aging-related lung function decline. Age.

